# Unveiling the Therapeutic Potential of “Taikong Blue” Lavender Essential Oil and Its Key Compounds in Skin Problems via Network Pharmacology and In Vitro Validation

**DOI:** 10.1111/jocd.70640

**Published:** 2026-01-28

**Authors:** Fei Liu, Yingyu Zhang, Minhazul Abedin, Jingyi Song, Suzhen Yang, Hongxiang Lou, Xixi Dou, Junsong Xiao, Hua Wu

**Affiliations:** ^1^ Shandong Provincial Key Laboratory of Carbohydrate and Glycoconjugate Drugs, Shandong Freda Pharmaceutical Group co., Ltd. Ji'nan Shandong China; ^2^ Department of Natural Product Chemistry, Key Laboratory of Natural Products & Chemical Biology of Ministry of Education, School of Pharmaceutical Sciences Shandong University Ji'nan China; ^3^ Beijing Technology and Business University Beijing China

**Keywords:** network pharmacology, skin barrier restoration, skin problem, taikong blue lavender essential oil, tNF signaling pathway

## Abstract

**Background:**

Taikong Blue Lavender Essential Oil (TLEO) is derived from a proprietary space‐bred cultivar of *Lavandula angustifolia* and cultivated under pristine conditions in Xinjiang, China. TLEO has long been used by regional people to treat various skin disorders such as hyperpigmentation, trans‐epidermal water loss, collagen degradation, and poor wound healing. Despite the ethnopharmacological applications of TLEO, the molecular basis of its dermatological efficacy remains poorly defined.

**Method:**

This study integrated network pharmacology, molecular docking, and in vitro assays to systematically investigate how TLEO works against inflammatory skin conditions, focusing on its key compounds and biological targets.

**Results:**

A total of 66 skin disorder‐related genes were identified through network pharmacology, with gene enrichment analyses highlighting the TNF signaling pathway as a critical mediator. Protein–protein interaction analysis revealed MMP9, EGFR, and PTGS2 as core targets. Molecular docking confirmed that linalool and linalyl acetate, the primary constituents of TLEO, exhibited moderate binding affinities with these targets. In vitro experiments using TNF‐*α*‐stimulated HaCaT cells demonstrated that treatment with 0.01% TLEO significantly (*p <* 0.05) reduced oxidative stress markers (NO, ROS, MDA), restored antioxidant enzymes (SOD, CAT), and downregulated inflammatory cytokines (IL‐6, IL‐1*β*, IL‐8). TLEO also inhibited the phosphorylation of p38 MAPK and NF‐*κ*B p65, suppressed PTGS2 and MMP9 expression, and restored EGFR levels, indicating anti‐inflammatory and barrier‐restorative functions.

**Conclusions:**

The study establishes a scientific foundation for the use of TLEO as a multifunctional ingredient in dermatological applications and highlights its value as a sustainable crop for regional economic development in Xinjiang.

## 1 Introduction

The skin is the largest and the primary barrier of the human body. Consequently, it is perpetually exposed to environmental stressors such as pollution, UV radiation, and microbial pathogens, which disrupt its structural integrity and homeostatic functions. These harmful factors can cause common skin problems like dryness, premature aging, dark spots, and sores [1, 2]. Over time, these issues weaken the skin's natural protective layer. A weakened skin barrier makes it easier to develop long‐term inflammation such as psoriasis and eczema. Inflammatory cytokines such as tumor necrosis factor‐*α* (TNF‐*α`s*) drive oxidative stress, collagen degradation via matrix metalloproteinases (MMPs), and aberrant cytokine signaling (e.g., IL‐6, IL‐1*β*) [3]. Ultraviolet exposure and oxidative stress also overstimulate melanocytes, causing uneven pigmentation and dark spots [4, 5]. Overproduction of sebum creates an ideal environment for acne‐causing bacteria, while a compromised barrier permits allergen entry, triggering immune responses that worsen atopic dermatitis [6, 7]. These visible symptoms often correlate with elevated levels of anxiety and depression, highlighting the close relationship between skin and mental health.

Lavender essential oil (LEO), derived from *Lavandula angustifolia* , has garnered significant industrial interest for its broad‐spectrum bioactivities, including anti‐inflammatory, antioxidant, and wound‐healing properties [8, 9, 10]. Its efficacy in mitigating oxidative stress, promoting collagen synthesis, and modulating immune responses has positioned it as a cornerstone ingredient in skincare formulations, aromatherapy, and pharmaceutical products [8, 11, 12]. The global essential oil market, estimated to reach 15 billion USD by 2025, reflects escalating demand for natural, sustainable alternatives to synthetic compounds, with LEO contributing substantially due to its versatility and consumer appeal [13]. However, conventional LEO faces limitations, such as variability in bioactive composition and insufficient mechanistic validation for multifunctional applications.

“Taikong Blue” lavender essential oil (TLEO) is derived from a novel lavender cultivar developed through space breeding in Xinjiang, China. This cultivar demonstrates notable improvements over conventional varieties, including a prolonged flowering period and higher essential oil yield. The resulting essential oil is characterized by a unique chemical profile, with significantly elevated concentrations of linalool (about 30%) and linalyl acetate (about 40%). Preliminary studies indicate that these compositional advantages enhance its anti‐inflammatory and antioxidant properties, underscoring the greater potential of “Taikong Blue” lavender essential oil for industrial applications [14].

While prior research highlights its capacity to suppress LPS‐induced inflammation via MAPK‐NF‐*κ*B signaling, its therapeutic mechanisms against skin pathologies remain unexplored. Furthermore, the ability of TLEO to concurrently address multiple dermatological disorders like dryness, aging, hyperpigmentation, and ulceration has not been systematically investigated, limiting its commercial optimization. And thus this study integrates network pharmacology, molecular docking, and in vitro validation to unravel TLEO's multi‐target mechanisms against TNF‐*α*‐mediated skin inflammation and barrier dysfunction. By identifying core targets (e.g., MMP9, EGFR, PTGS2) and pathways (TNF, NF‐*κ*B, MAPK), we aim to establish a mechanistic foundation for the application of TLEO in skincare therapeutics.

## 2 Materials and Methods

### 2.1 Chemical Reagents

TLEO (≥ 98% purity, HPLC‐verified) was generously provided by Eprhan Spices Co. Ltd. (Xinjiang Autonomous Region, China). To ensure experimental consistency, the same batch of TLEO was used throughout the study. Working solutions were freshly prepared from stock prior to each experiment using a standardized dilution protocol to maintain accurate concentrations and ensure reproducibility. Cell culture reagents included DMEM and penicillin/streptomycin (Eallbio, Beijing, China), fetal bovine serum (FBS; FuHeng, Shanghai, China), and CCK‐8 solution (Biosharp, Beijing, China). Recombinant human TNF‐*α* (catalog no. H8916) was purchased from Sigma–Aldrich (St. Louis, MO, USA).

Assay kits for reactive oxygen species (ROS), nitric oxide (NO; S0023), catalase (CAT), superoxide dismutase (SOD), malondialdehyde (MDA), and the BCA protein quantification kit were obtained from Beyotime Biotechnology (Shanghai, China). 2′,7′‐dichlorodihydrofluorescein diacetate (DCFH‐DA) was also purchased from Beyotime Biotechnology. Rabbit‐derived primary antibodies targeting *β*‐actin, GAPDH, MMP9, EGFR, and PTGS2 were sourced from Cell Signaling Technology (Danvers, MA, USA). Gene‐specific primers for *β*‐actin, IL‐6, IL‐1*β*, IL‐8, MMP9, EGFR, and PTGS2 were synthesized by the Beijing Genomics Institute (Beijing, China). RNA extraction kits were procured from TransGen Biotech (Beijing, China), SYBR Green Master Mix from TOYOBO (Japan), total protein extraction kits from Nanjing Jincheng Bioengineering Institute (Nanjing, China), and ultrasensitive ECL substrate from Tanon (Shanghai, China). All additional analytical‐grade chemicals were supplied by Beijing Chemical Works (Beijing, China).

### 2.2 Predicting Targets of TLEO and Skin Problems‐Related Genes

In previous work, 53 compounds within TLEO were identified via Gas Chromatography–Mass Spectrometry (GC–MS). These compounds were validated using the Traditional Chinese Medicine Systems Pharmacology Database and Analysis Platform (TCMSP) and PubChem. Pharmacokinetic properties, including oral bioavailability and drug‐likeness (DL), were assessed via SwissADMET to prioritize bioactive constituents [15, 16]. Simplified Molecular Input Line Entry System (SMILES) notations of these compounds were input into the SwissTargetPrediction tool to predict putative human ( *Homo sapiens* ) molecular targets [17].

Target genes associated with cutaneous conditions, for example, dryness, premature aging, ulceration, and hyperpigmentation, were collated from GeneCards and the NCBI Disease Gene Database [18]. The Venny 2.0 tool was utilized to identify overlapping target genes linked to these dermatological pathologies, generating Venn diagrams to visualize shared targets between TLEO and the four‐skin condition (https://bioinfogp.cnb.csic.es/tools/venny/index.html). Gene Ontology (GO) and Kyoto Encyclopedia of Genes and Genomes (KEGG) pathway enrichment analyses were performed using WebGestalt and KOBAS 3.0 (*p <* 0.05) to elucidate biological functions, molecular mechanisms, and key regulatory pathways [19, 20].

### 2.3 Protein–Protein Interaction Network Analysis

The identified common targets were validated using UniProtKB for protein name annotation and subsequently analyzed for protein–protein interactions (PPIs) via the STRING database (https://string‐db.org). A high‐confidence interaction threshold (> 0.9) was applied to ensure robust associations. The resultant PPI network data were downloaded as a TSV file and imported into Cytoscape software (version 3.9.1; Cytoscape Consortium, Boston, MA, USA) for network visualization and analysis. Hub genes were identified using CytoHubba by evaluating interaction density and centrality metrics, including degree centrality (DC), betweenness centrality (BC), and closeness centrality (CC). These parameters enabled the systematic prioritization of potential core targets based on their topological significance and influence within the network [21].

### 2.4 Molecular Docking

The top 12 candidate compounds and corresponding protein targets were selected for molecular docking based on interaction scores derived from compound‐target network analysis.

#### 2.4.1 Ligand Preparation

Small‐molecule ligand structures were retrieved in SDF format from PubChem and energy‐minimized using the Merck Molecular Force Field 94 (MMFF94). Structural optimization was performed in Avogadro (v1.2.0) under physiological pH conditions (7.0), followed by conversion to PDBQT file format via Open Babel to ensure compatibility with docking software.

#### 2.4.2 Protein Preparation

Protein sequences were acquired from the UniProtKB database, and homologous structures were identified using Position‐Specific Iterated BLAST (PSI‐BLAST) against the Protein Data Bank (PDB). The highest‐confidence structural homologs were selected based on sequence similarity and E‐value thresholds. Energy minimization of the protein structures was conducted using Swiss‐PDB, followed by removal of non‐essential water molecules and co‐crystallized ligands. Polar hydrogen atoms were added to the protein structures using AutoDock Tools (v1.5.6), and the final structures were converted to PDBQT format. The active binding site of each target protein was computationally predicted using the CASTp v3.0 server (Computed Atlas of Surface Topography of Proteins; http://sts.bioe.uic.edu/castp), employing geometric and topological criteria as previously described.

#### 2.4.3 Molecular Docking

Ligand‐receptor docking was performed using AutoDock Vina (v1.1.2) with a grid box centered on the predicted active site. Ten ligand conformations were generated per docking simulation, and the optimal pose was selected based on calculated binding affinity (kcal/mol) and spatial complementarity. Molecular interactions and conformational analyses were visualized and validated using PyMOL (v2.5.7) and Discovery Studio 2019 (v19.1.0).

### 2.5 Cell Culture and Cell Viability Assay

The immortalized human keratinocyte cell line (HaCaT) was procured from FuHeng Cell Center (Shanghai, China). Cells were maintained in Dulbecco's Modified Eagle Medium (DMEM) supplemented with 10% fetal bovine serum (FBS, v/v) and 1% penicillin–streptomycin (P/S) at 37°C under a humidified 5% CO2 atmosphere. Subculturing was performed at 80%–90% confluence using standard trypsinization protocols, with culture conditions remaining consistent throughout the study unless otherwise specified.

For viability assessment, HaCaT cells were seeded into 96‐well plates at a density of 2 × 10^4^ cells/well and allowed to adhere for 24 h under standard conditions. Cells were then stimulated with 20 ng/mL recombinant human TNF‐*α* (Sigma‐Aldrich, H8916) for 12 h to induce an inflammatory response. Subsequently, the medium was replaced with fresh DMEM containing 0.001%–0.1% (v/v) TLEO (diluted in fresh medium) for an additional 12‐h incubation.

Cell viability was quantified using the CCK‐8 assay (Biosharp, Beijing, China). Briefly, 100 μL of serum‐free DMEM containing 10% (v/v) CCK‐8 reagent was added to each well, followed by incubation at 37°C for 30 min. Absorbance was measured at 450 nm using an Infinite 200 Pro microplate reader (Tecan, Männedorf, Switzerland). Viability was calculated as:

$$\text{Cell viability}\left(\%\right)=\frac{\left(\text{Absorbance of sample}\right)}{\left(\text{Absorbance of control}\right)}\times 100\%$$

### 2.6 Oxidative Stress and Biochemical Assay

Following a 12‐h treatment with 0.001%–0.01% (v/v) TLEO, cell culture supernatants from TNF‐*α*‐stimulated HaCaT cells (2 × 10^4^ cells/well) were collected for NO analysis. NO levels were determined using a commercial NO assay kit according to the manufacturer's protocol. Briefly, standard solutions and samples were mixed with assay reagents in triplicate and incubated at 37°C for 30 min. Absorbance was measured at 540 nm using an Infinite 200 Pro microplate reader (Tecan, Männedorf, Switzerland). Following the same treatments as the NO assay, cells were washed twice with phosphate‐buffered saline (PBS, pH 7.4) and incubated with 10 μM DCFH‐DA in serum‐free DMEM at 37°C for 30 min. After three washes with serum‐free medium, fluorescence intensity was quantified at excitation/emission wavelengths of 485/535 nm using a fluorescence microplate reader.

HaCaT cells (1.5 × 10^6^ cells/60 mm dish) were pre‐treated with 20 ng/mL TNF‐*α* for 12 h, followed by TLEO (0.001%–0.01%) for 12 h. Cells were lysed in ice‐cold RIPA buffer (Beyotime Biotechnology) after twice PBS washes. Lysates were centrifuged at 10000 × g for 5 min at 4°C, and supernatants were collected. MDA, SOD, and CAT activities were quantified using commercial assay kits according to manufacturer guidelines.

### 2.7 Quantitative Real‐Time Polymerase Chain Reaction (qRT–PCR)

HaCaT cells were seeded in 60 mm culture dishes at a density of 1.5 × 10^6^ cells/dish and treated with 20 ng/mL TNF‐*α* in combination with 0.001%–0.01% (v/v) TLEO for 12 h after reaching confluence. Total RNA was extracted using the TransZol Up Plus RNA Kit. RNA purity and concentration were assessed using a NanoDrop q3000 spectrophotometer (Thermo Fisher Scientific, Waltham, MA, USA), with RNA integrity confirmed by an A260/A280 absorbance ratio of 1.8–2.1. For cDNA synthesis, 500 ng of total RNA was reverse‐transcribed using ReverTra Ace qPCR RT Kit (Toyobo Co. Ltd., Japan). Quantitative real‐time PCR (qRT‐PCR) was performed on a BioRad CFX96 system (BioRad, USA) under the following cycling conditions: initial denaturation at 95°C for 2 min; 38 cycles of denaturation at 95°C for 30 s, annealing at 57°C for 30 s, and extension at 72°C for 30 s; followed by a final extension at 72°C for 2 min. *β*‐actin was used as an endogenous reference gene to normalize target gene expression levels, and relative quantification was performed using the 2^−ΔΔCt^ method [22]. The primer list had been presented in Table 1.

**TABLE 1:** Primer sequences for RT‐PCR.

Primer	Forward	Reverse
*β*‐Actin	CCTAGAAGCATTTGCGGTGCACGATG	TCATGAAGTGTGACGTTGACATCCGT
TNFα	TGGCGTGGAGCTGAGAGATAACC	GACGGCGATGCGGCTGATG
IL‐6	CACTGGTCTTTTGGAGTTTGAG	GGACTTTTGTACTCATCTGCAC
IL‐8	TTATGAATTCTCAGCCCTCTTCAAAAACTTCTC	ATGACTTCCAAGCTGGCCGTG
IL‐1*β*	CTGTAGTGGTGGTCGGAGATTCG	CAGTGGCAATGAGGATGACTTGTTC
EGFR	ACCCATATGTACCATCGATGTC	GAATTCGATGATCAACTCACGG
MMP9	CAGTACCGAGAGAAAGCCTATT	CAGGATGTCATAGGTCACGTAG
PTGS2	TGTCAAAACCGAGGTGTATGTA	AACGTTCCAAAATCCCTTGAAG

### 2.8 Western Blot Analysis

After TNF‐*α* treatment on the cells (1.5 × 10^6^ cells/dish), the medium was replaced with fresh culture medium containing 0.01% (v/v) TLEO for an additional 12 h. Cells were lysed with 300 μL of ice‐cold RIPA buffer supplemented with phosphatase and protease inhibitor cocktails (Roche, Switzerland). Lysates were centrifuged at 10000 × g for 10 min at 4°C, and the supernatant was collected. Total protein concentration was determined using a BCA assay kit and samples were adjusted to equal concentrations for downstream analysis. Protein samples were resolved on 13% SDS‐PAGE gels (5% stacking gel) and transferred to PVDF membranes (Merck KGaA, Germany). Membranes were blocked with 5% non‐fat milk in TBST for 2 h at room temperature, followed by incubation with primary antibodies (diluted in TBST) for 2 h at room temperature. After three washes with TBST (Biosharp, China), membranes were incubated with HRP‐conjugated anti‐rabbit IgG secondary antibodies (1:1000; Cell Signaling Technology, USA) for 1 h. Following additional TBST washes, protein bands were visualized using an enhanced chemiluminescence (ECL) substrate (Tecan, Switzerland). Band intensities were quantified using ImageJ software (NIH, USA), and target protein expression levels were normalized to *β*‐actin as a loading control.

### 2.9 Pharmacophore Analysis and Double Docking

Two bioactive constituents of TLEO were selected for further evaluation based on their optimal ADMET properties, binding affinity scores, and relative abundance (quantified via GC–MS analysis). To investigate potential multi‐target binding mechanisms, sequential docking was performed using the CB‐Dock server, a computational tool for ensemble docking and alternative binding pocket identification. For this step, the protein‐ligand complex exhibiting the highest binding affinity (from prior molecular docking results) was designated as the receptor structure. A secondary ligand was then docked into the receptor‐ligand complex using CB‐Dock to evaluate synergistic interactions or competitive binding at distinct sites.

### 2.10 Statistical Analysis

All statistical analyses were performed using IBM SPSS Statistics (version 22.0). One‐way ANOVA was applied to assess differences among groups, followed by the Duncan's *post hoc* test to assign alphabetical significance markers (*p <* 0.05). Data visualization and graphical representations were generated using GraphPad Prism (version 9.0).

## 3 Results

### 3.1 Predicted Targets of TLEO and Skin Disease‐Related Genes

**FIGURE 1:**
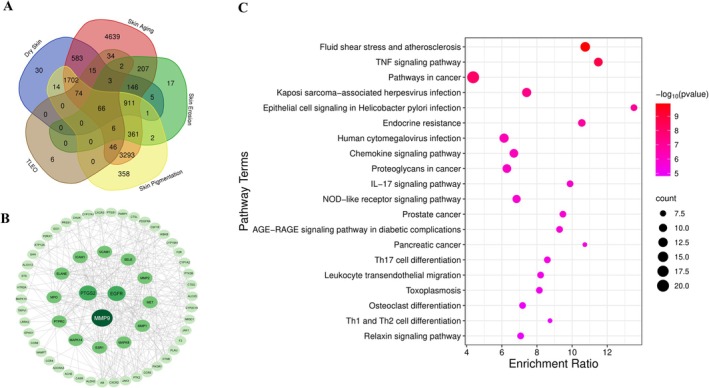
Network pharmacology analysis of TLEO targets. (A) Venn diagram of TLEO components, Dry Skin, Skin Aging, Skin Erosion, and Skin Pigmentation effects. (B) STRING network of protein–protein interactions. (C) Enrichment ratio of KEGG pathways involved with those genes.

Based on the ADMET analysis results, 53 identified components of TLEO were further refined on the basis of criteria for oral bioavailability and drug‐like properties, leading to the selection of 21 key active components (see Data S2: Table S2). These included 5 monoterpenes, 3 sesquiterpenes, 3 diterpenes, 4 alcohols, and 5 esters. 256 targets were predicted for these 21 key compounds from Swiss‐Target prediction. After subsequent non‐coding genes were eliminated for skin pathologies related targets, 3550 genes, 12 088 genes, 1727 genes and 6834 were found to be associated with dryness, aging, ulceration, and hyperpigmentation. Using the Venny 2.0 online tool, a comprehensive integration of target genes derived from active components and four skin‐related disorders led to the successful identification of 66 common key target genes (Figure 1A).

### 3.2 GO And KEGG Enrichment Analysis

GO and KEGG enrichment analyses were subsequently performed on these 66 genes, which demonstrated significant relevance in mitigating skin disorders. GO analysis indicated that these genes are predominantly enriched in 12 biological processes, including cellular metabolism, response to stimuli, and biological regulation. Additionally, these genes are associated with 18 cellular components, such as the cell membrane and nucleus, and 17 molecular functions related to protein binding and ion binding (see Data S1, Figure S1).

The KEGG analysis suggested that TLEO may predominantly alleviate four prevalent skin conditions through several pathways, including blood flow shear stress, atherosclerosis, and the TNF signaling pathway (Figure 1C and Data S2, Table S4). Notably, the TNF signaling pathway has emerged as particularly significant, given its activation in various dermatological diseases. TNF‐*α* signaling is involved in the activation of the NF‐*κ*B and MAPK cascades, both of which are crucial for the TNF signaling pathway. Therefore, this process involves not only the expression of PTGS2 and MMP9 but also the phosphorylation of NF‐*κ*B and MAPK. Modulating these targets may potentially mitigate dermatological pathologies. Thus, we propose that the potential mechanism of action of TLEO may be associated with its impact on NF‐*κ*B/MAPK, PTGS2, and MMP9 in the TNF signaling pathway.

### 3.3 PPI Network Analysis

The resulting network consisted of 65 nodes and 792 edges (Figure 1B). Further analysis using the CytoNCA plugin identified core targets based on degree, betweenness, and closeness centrality metrics. Key targets implicated in therapeutic effects of TLEO against the four skin conditions included MMP9, EGFR, PTGS2, VCAM1, ESR1, PTPRC, ICAM1, MMP1, MMP2, SELE, MAPK14, and MAPK8. Among these, MMP9, EGFR, and PTGS2 ranked consistently within the top three across all centrality metrics, highlighting their pivotal roles as potential therapeutic hubs (see Data S1 and S2, Figure S1 and Table S2).

### 3.4 Molecular Docking Simulation of Human Hub Genes

**FIGURE 2:**
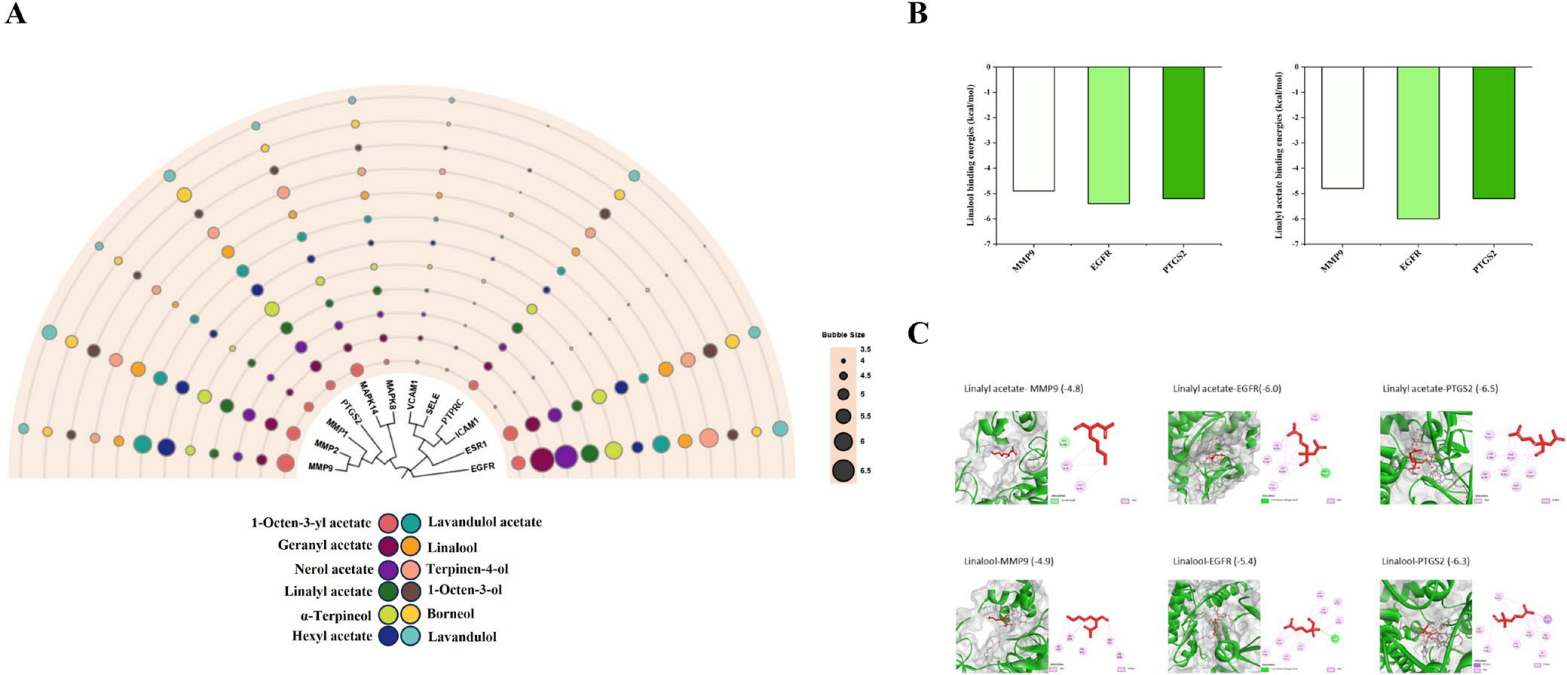
Interactions between TLEO active components as ligands and target proteins. (A) Binding affinities between active components and key targets. (B) The binding energy results of molecular docking of Linalool and Linalyl acetate. (C) 2D and 3D interaction effects between potential key targets: EGFR, MMP9, PTGS2, and main active components: Linalool and linalyl acetate.

The selected protein sequence ID and their location were provided in Data S2, Table S3. Molecular docking was performed using AutoDock Vina (version 1.2.0) to evaluate ligand‐target interactions. As illustrated in Figure 2A–C, binding affinities between TLEO components and target proteins exhibited binding energies below −4.5 kcal/mol, suggesting potentially stable and favorable interactions. The core therapeutic targets, MMP9, EGFR, and PTGS2, demonstrated prominent binding (≤ −4.5 kcal/mol) with the top 12 TLEO components, achieving an average binding energy of −5.15 kcal/mol. These results highlight their candidate role in mediating TLEO's protective mechanisms against skin disorders (see Data S1 and S2, Figures S3–S5 and Table S5).

Linalool (29.48%) and linalyl acetate (40.97%), the predominant constituents of TLEO (collectively > 70% of its composition), displayed discernible binding with all three core targets. Linalyl acetate exhibited binding energies of −6.0, −4.8, and −5.2 kcal/mol with EGFR, MMP9, and PTGS2, respectively, while linalool showed values of −5.4, −4.9, and −5.2 kcal/mol (Figure 2). A detailed summary of binding energies for the 12 prioritized TLEO components is provided in Table S2 (Data S2).

### 3.5 Cell Model Construction

**FIGURE 3:**
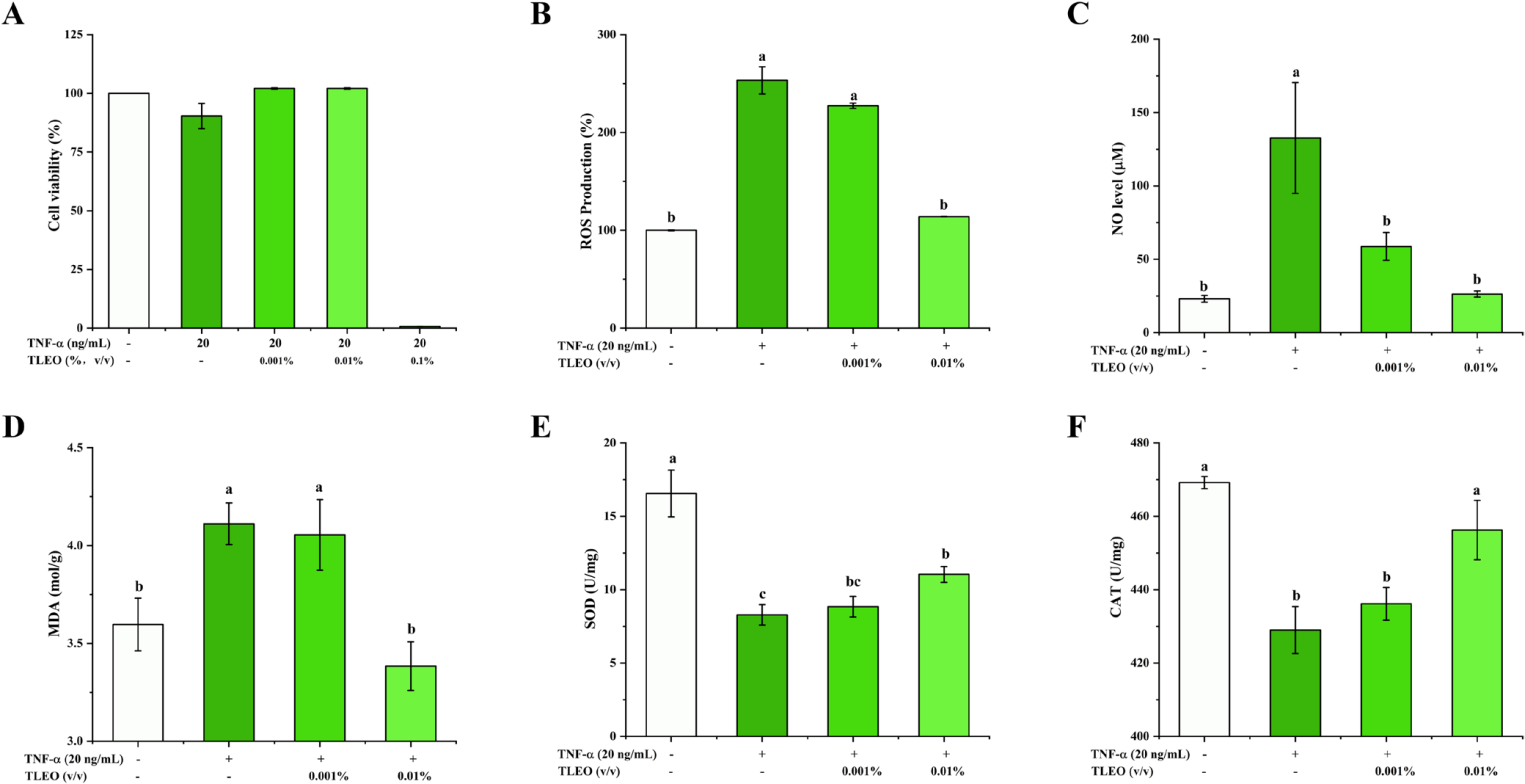
Effects of TNF‐*α* and TLEO on cell viability and oxidative stress markers of HaCaT. (A) Impact of TLEO on TNF‐*α*‐induced HaCaT cell viability; (B) The intracellular reactive ROS level; (C) The content of NO in cell culture medium; (D) The intracellular reactive MDA level; (E, F) Impact of TLEO on SOD and CAT activities in TNF‐*α*‐induced HaCaT cells. Waller‐Duncan was used for posthoc analysis and “a,b,c” denotes the significance between groups (*p* < 0.05).

The impact of TNF‐*α* (20–60 ng/mL) on HaCaT cell viability and inflammatory cytokine expression was assessed using CCK‐8 assays and quantitative real‐time PCR (qRT‐PCR). TNF‐*α* treatment did not significantly reduce cell viability (survival rates > 90% across all concentrations) but markedly upregulated the transcription of pro‐inflammatory cytokines IL‐6, IL‐1*β*, and IL‐8 (see Data S1, Figure S6). Based on these results, 20 ng/mL TNF‐*α* was selected to establish the HaCaT inflammatory injury model. CCK‐8 assays revealed that co‐treatment with TLEO (0.001–0.1%, v/v) and TNF‐*α* (20 ng/mL) did not compromise cell viability at 0.001–0.01% TLEO. However, 0.1% TLEO induced significant cytotoxicity (viability < 80%; Figure 3A). Consequently, 0.001% and 0.01% TLEO were chosen for subsequent mechanistic studies.

### 3.6 Effects of TLEO on the Oxidative Stress Response of HaCaT Cells Treated With TNF‐*α*

NO and ROS are critical mediators of inflammation and drivers of oxidative stress. Excessive MDA production disrupts cellular redox homeostasis by overwhelming endogenous free radical scavenging systems, exacerbating oxidative damage. As shown in Figure 3B–D, treatment with 20 ng/mL TNF‐*α* significantly elevated NO, ROS, and MDA levels. Co‐treatment with 0.01% TLEO (v/v) effectively attenuated these increases, restoring all three markers to near‐baseline levels (*p* < 0.05 vs. TNF‐*α* group).

TNF‐*α* suppressed the activity of SOD and CAT, pivotal antioxidant enzymes that neutralize free radicals. TLEO co‐treatment dose‐dependently reversed this suppression. 0.001% TLEO partially rescued enzymatic activity, while 0.01% TLEO significantly restored SOD activity (*p* < 0.05 vs. TNF‐*α*) and normalized CAT activity to near‐control levels (Figure 3E,F).

### 3.7 Effects of TLEO on TNF‐*α*‐Treated HaCaT Inflammatory Cytokines and Target Gene Expression

**FIGURE 4:**
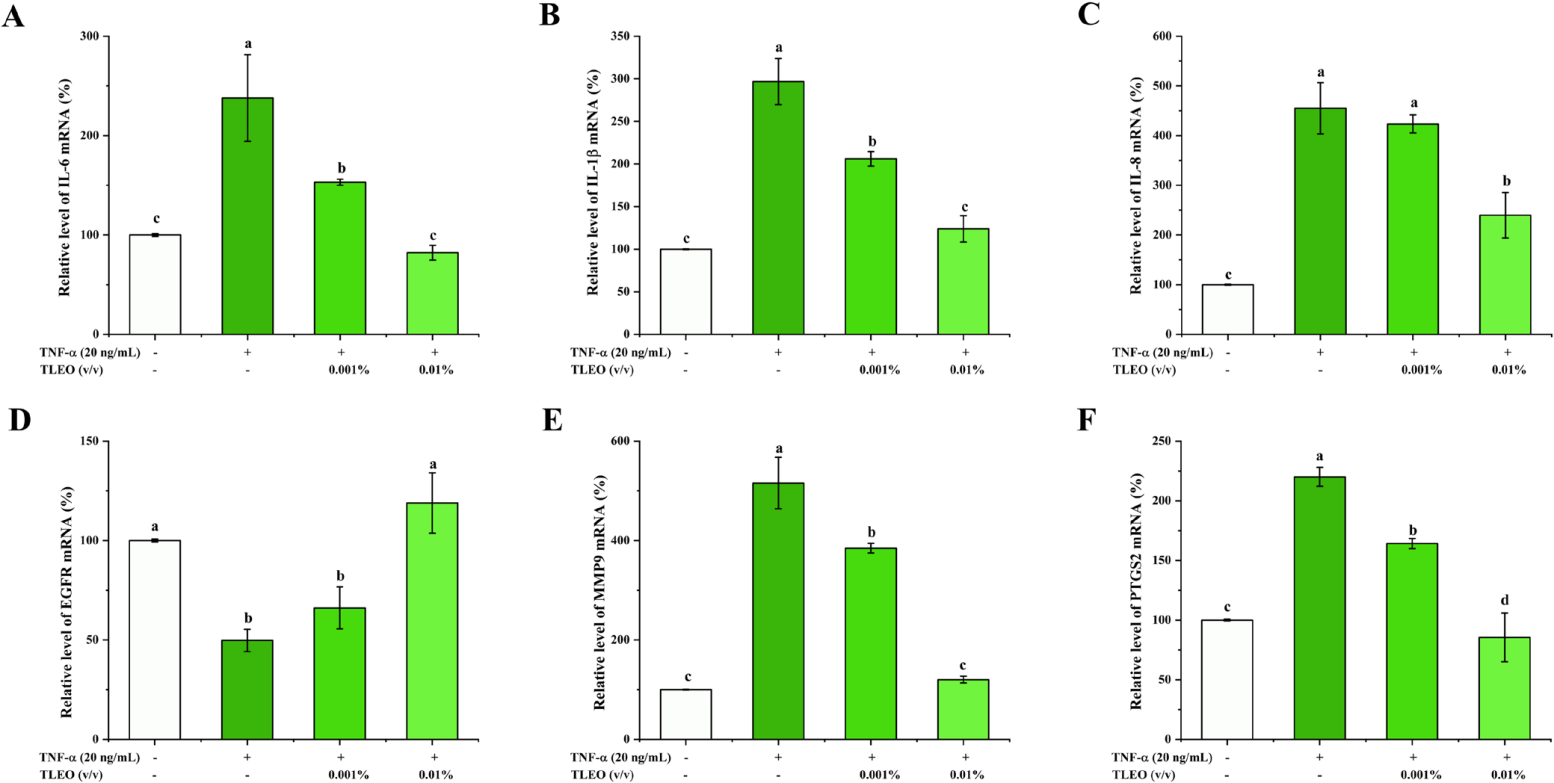
Effects of TLEO on TNF‐*α*‐induced proinflammatory cytokines and key targets. (A–C) The effect of TLEO on the mRNA expression of IL‐6, IL‐1*β*, and IL‐8 in HaCaT cells induced by TNF‐*α*. (D–F) The effect of TLEO on the mRNA expression of EGFR, MMP9, and PTGS2 in TNF‐*α*‐induced HaCaT cells. Waller‐Duncan was used for posthoc analysis and “a,b,c” denotes the significance between groups (*p* < 0.05).

As shown in Figure 4A–C, TLEO significantly suppressed TNF‐*α*‐induced mRNA expression of pro‐inflammatory cytokines IL‐6, IL‐1*β*, and IL‐8 in HaCaT cells (*p* < 0.05 vs. TNF‐*α* group). At 0.01% (v/v), TLEO restored IL‐6 and IL‐1*β* transcript levels to near‐baseline (control group) and reduced IL‐8 expression to 40%–50% of the TNF‐*α*‐induced model. In contrast, 0.001% TLEO only partially attenuated IL‐8 expression (60%–70% of TNF‐α group; *p <* 0.05), with no significant impact on IL‐6 or IL‐1*β*.

TNF‐*α* stimulation dysregulated key genes identified in the PPI network: EGFR expression was downregulated, while PTGS2 and MMP9 were upregulated. Co‐treatment with 0.01% TLEO normalized EGFR to control levels, suppressed PTGS2 below baseline (*p* < 0.05), and restored MMP9 expression to near‐control levels (*p* < 0.05). Conversely, 0.001% TLEO only partially attenuated MMP9 and PTGS2 (*p* < 0.05) and showed no significant effect on EGFR (Figure 4D–F).

### 3.8 Effects of TLEO and Its Main Components on the Expression Levels of Target Proteins

**FIGURE 5:**
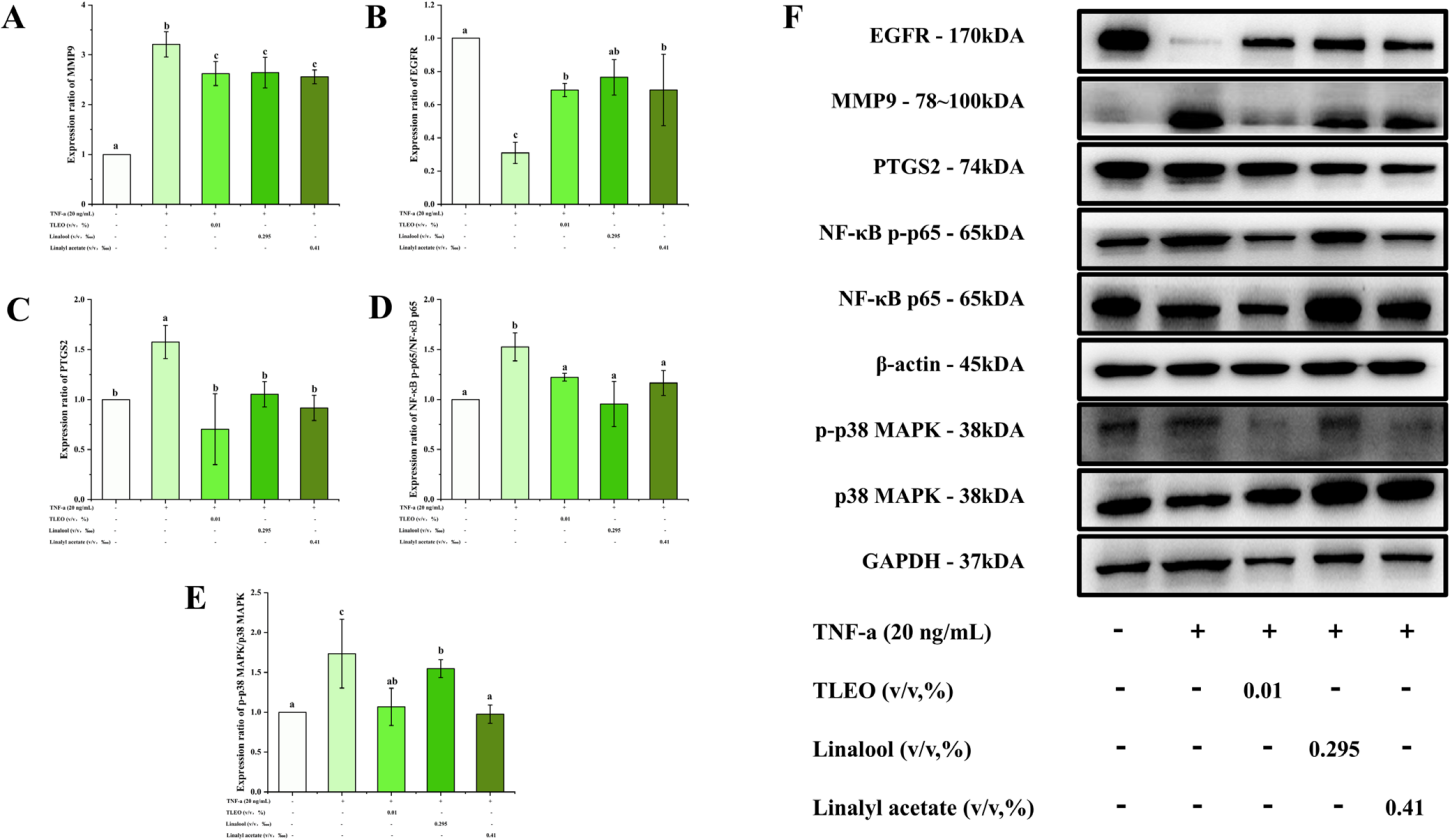
Effects of TLEO on the expression of key proteins involved in TNF‐*α*‐induced activation of HaCaT cells. (A–C) The effect of TLEO on the expression of MMP9, EGFR, and PTGS2 proteins in HaCaT cells induced by TNF‐*α*. (D, E) The effect of TLEO on the expression and phosphorylation levels of p38 MAPK and NF‐*κ*B p65 proteins in HaCaT cells induced by TNF‐*α*. (F) Western blot bands for the proteins. Waller‐Duncan was used for post hoc analysis and “a,b,c” denotes the significance between groups (*p* < 0.05).

Based on its experimental efficacy, a 0.01% (v/v) TLEO formulation, comprising linalool (29.48%) and linalyl acetate (40.97%) as determined by GC–MS, was selected for mechanistic investigation. To evaluate the effects of individual components, linalool and linalyl acetate were administered at concentrations reflecting their proportional presence in the TLEO formulation. As demonstrated in Figure 5, both TLEO and its components significantly suppressed TNF‐*α*‐induced activation of key inflammatory mediators. Specifically, treatment resulted in marked reductions in phosphorylated p38 MAPK (p‐p38/p38) and NF‐*κ*B p65 (p‐p65/p65) levels, as well as decreases in PTGS2 and MMP9 expression compared to the TNF‐*α*‐treated group. Notably, TLEO and linalyl acetate showed superior inhibition of p38 MAPK signaling, and both restored PTGS2 and p‐p65/p65 levels to near‐baseline values (*p* < 0.05). Additionally, the TNF‐*α*‐induced suppression of EGFR was reversed by TLEO, linalool, and linalyl acetate (*p* < 0.05 vs. the TNF‐*α* group), with linalool being the most effective at restoring EGFR expression to control levels. These findings indicate that while linalyl acetate exhibits stronger anti‐inflammatory activity through suppression of the p38/NF‐*κ*B axis, linalool uniquely rescues EGFR function. Collectively, the anti‐inflammatory effects of TLEO in HaCaT cells appear to be mediated by the inhibition of p38 MAPK/NF‐*κ*B activation, resulting in reduced expression of downstream effectors such as PTGS2 and MMP9, and by the restoration of EGFR signaling to counteract TNF‐*α*‐induced epidermal dysfunction. All the uncropped gel images had been provided in Data S3.

### 3.9 Double Docking Analysis and Their Interactions

**FIGURE 6:**
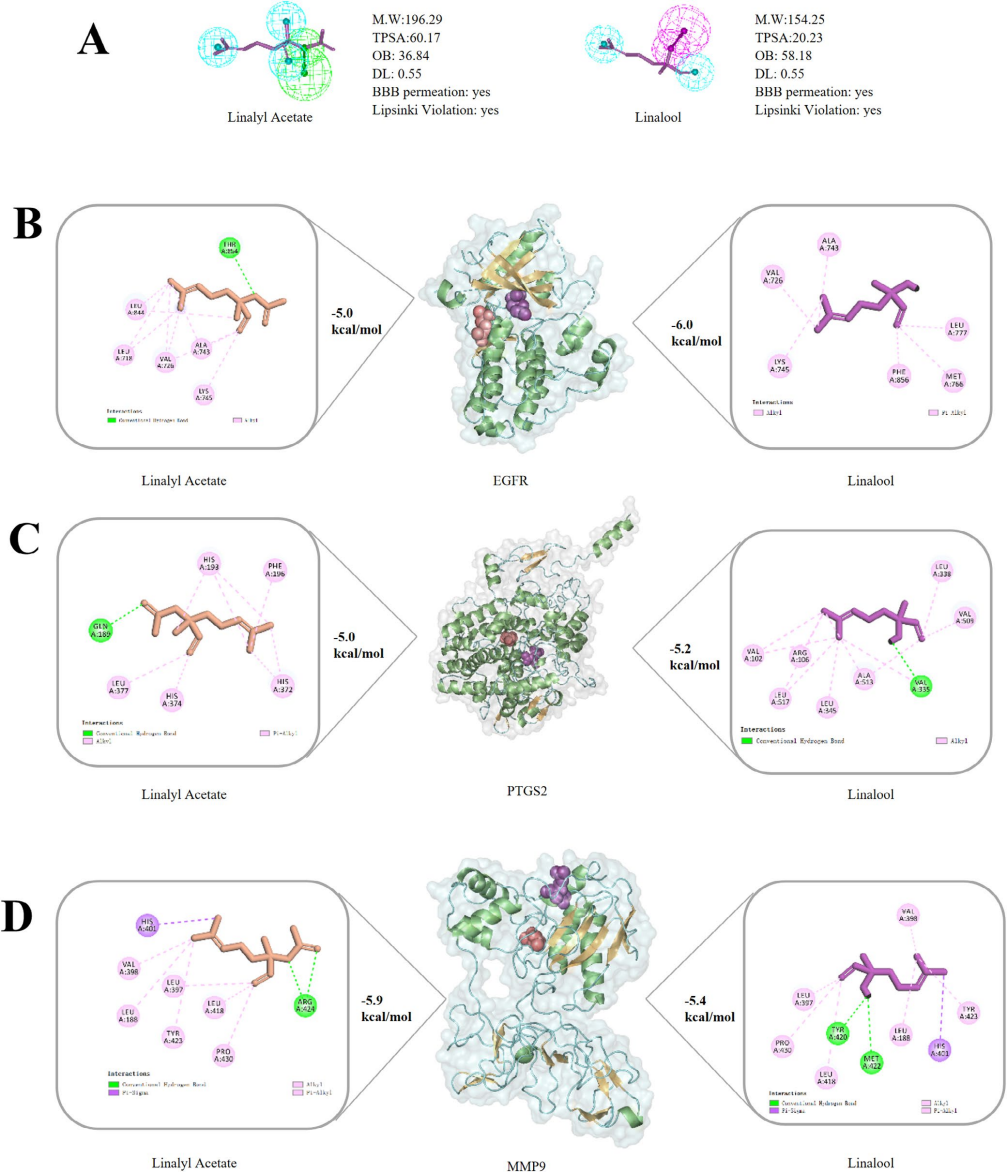
The double docking interactions between linalyl acetate‐proteins‐linalool. (A) Pharmacophore profile of linalool and linalyl acetate; (B) Linalyl acetate‐EGFR‐Linalool complex; (C) Linalyl acetate‐PTGS2‐Linalool complex; (D) Linalyl acetate‐MMP9‐Linalool complex.

Based on molecular docking results in 3.4 section, linalyl acetate exhibited the strongest binding affinities to EGFR (−6.0 kcal/mol), MMP9 (−5.2 kcal/mol), and PTGS2 (−5.2 kcal/mol). To evaluate synergistic interactions, linalool was subsequently docked into these preformed linalyl acetate‐protein complexes. Blind docking revealed moderate binding energies for linalool: −5.0 kcal/mol with the linalyl acetate‐EGFR complex, −4.9 kcal/mol with the linalyl acetate‐MMP9 complex, and −5.1 kcal/mol with the linalyl acetate‐PTGS2 complex (Figure 6). Analysis of interaction pockets (Table 2) identified critical residues stabilizing these complexes, including EGFR's ARG98, CYS99, and LEU397; MMP9's CYS21, HIS24, and PRO25; and PTGS2's PRO741, MET793, and LYS846. Notably, linalyl acetate and linalool shared overlapping binding regions in EGFR and PTGS2 but diverged in MMP9, suggesting compound‐specific interactions within the catalytic domains.

**TABLE 2:** Binding affinities and interaction pocket for double docking complexes.

Linalyl Acetate			Linalool	
Pocket	Affinity (kcal/mol)	Protein	Affinity (kcal/mol)	Pocket
THR37 ASN38 LEU39 LEU44 GLU47 TYR48 ARG51 TYR52 MET94 ARG95 THR96 PRO97 ARG98 ASP182 LYS184 ASP185 GLY186 LEU187	−5.4	EGFR	−4.9	ARG98 CYS99 LEU188 LEU397 VAL398 HIS401 GLU402 PRO415 GLU416 ALA417 LEU418 MET419 TYR420 PRO421 MET422 TYR423 ARG424 PHE425 THR426 GLU427 GLY428 PRO429 PRO430 LEU431 HIS432
LEU718 GLY719 SER720 GLY721 VAL726 ALA743 ILE744 LYS745 MET766 CYS775 ARG776 LEU777 LEU788 THR790 GLN791 LEU792 MET793 GLY796 CYS797 ASP800 ARG841 ASN842 LEU844 THR854 ASP855 PHE856 LEU858 MET1002	−6	MMP9	−5	CYS21 HIS24 PRO25 CYS26 GLN27 ASN28 ARG29 GLY30 VAL31 CYS32 ASP111 PRO113 PRO114 THR115 TYR116 GLY121 LYS123 SER124 TRP125 ALA127 PHE128 ALA137 LEU138 PRO139 PRO140 PRO142 GLN447 GLU451 LYS454 ARG455
PRO69 ASN72 VAL74 ILE77 LEU78 TRP85 ILE98 MET99 TYR101 VAL102 SER105 ARG106 VAL335 LEU338 SER339 TYR341 LEU345 PHE367 LEU370 TYR371 TRP373 ARG453 LYS459 PRO460 TYR461 GLU466 LEU467 GLU496 LYS497 PRO498 ARG499 PHE504 GLY505 GLU506 MET508 VAL509 GLU510 GLY512 ALA513 SER516 LEU517	−5.2	PTGS2	−5.1	PRO741 LEU792 MET793 PRO794 PHE795 LYS846 THR847 SER995 ASN996 TYR998 ARG999 MET1002 ASP1003 GLU1004 VAL1010 ASP1012

## 4 Discussion

**FIGURE 7:**
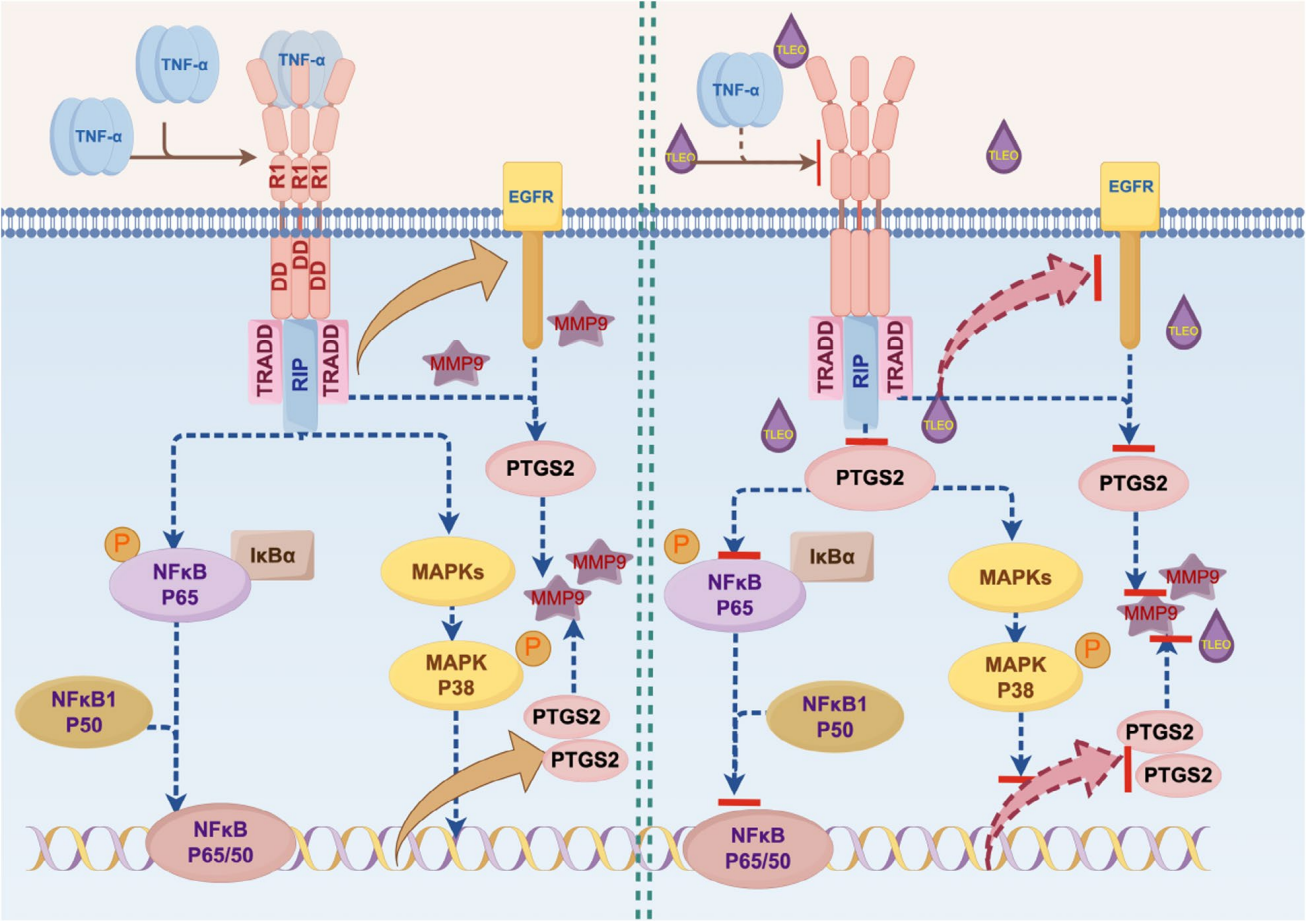
A schematic of the proposed mechanism by which TLEO inhibits TNF‐*α*‐stimulated inflammation in HaCaT cells.

This work integrated network pharmacology, molecular docking and in vitro assays to demonstrate the therapeutic capacity of TLEO against TNF‐*α*‐induced inflammation and barrier dysfunction in HaCaT cells. The results revealed that TLEO at a concentration of 0.01% (v/v) exerts simultaneous inhibition of inflammatory cascades, attenuation of oxidative damage and promotion of epidermal repair. Such a concerted action profile addresses multiple pathogenic features of inflammatory skin conditions and underscores the value of multi‐target natural products in dermatological applications Figure 7.

Network pharmacology mapping revealed 66 common targets linking TLEO bioactive constituents to skin disorders including hyperpigmentation, premature aging, and barrier impairment. The identification of MMP9, EGFR, and PTGS2 as core therapeutic targets in PPI aligns with their established roles in collagen turnover, keratinocyte proliferation, and prostaglandin‐mediated inflammation [23, 24, 25]. This multi‐target profile of TLEO contrasts sharply with single‐agent therapies. For instance, while doxycycline inhibits MMP9, its chronic use may be associated with increased oxidative stress. Conversely, biologics such as adalimumab effectively reduce inflammation by targeting TNF‐α, though their influence on EGFR‐driven re‐epithelialization remains less clearly established [26, 27, 28]. Low effective dose of TLEO (0.01%) aligns with trends favoring potent, low‐concentration natural products to minimize irritancy [29].

Molecular docking provided structural insights into the dual efficacy of major constituents. Linalyl acetate, representing 40.97% of TLEO composition, exhibited the highest binding affinities toward EGFR (−6.0 kcal/mol), MMP9 (−5.2 kcal/mol), and PTGS2 (−5.2 kcal/mol). Its interaction with the catalytic zinc‐binding triad of MMP9 (CYS21, HIS24, PRO25) suggests a mechanism for inhibition of collagen degradation, consistent with reduced MMP9 expression in functional assays. Linalool, present at 29.48%, showed preferential stabilization of EGFR via ARG98 and LEU397, providing a molecular explanation for restored receptor levels that support keratinocyte migration and re‐epithelialization. These residue‐specific interactions parallel findings in psoriasis models, where synergistic anti‐inflammatory and regenerative actions of linalool and linalyl acetate were demonstrated [30].

Experimental validation in TNF‐*α*‐stimulated HaCaT cells demonstrated that TLEO significantly reduces inflammation. Quantitative RT‐PCR (qRT‐PCR) analysis revealed dose‐dependent decreases in pro‐inflammatory cytokine mRNA levels (IL‐6, IL‐1*β*, IL‐8). Western Blot results confirmed that 0.01% TLEO suppressed p38 MAPK and NF‐*κ*B signaling, restoring phosphorylation levels of p38 and NF‐*κ*B p65 to near‐baseline states (Figure 6). These effects occurred at a tenfold lower concentration than conventional *Lavandula angustifolia* oils, which require > 0.1% to achieve comparable cytokine suppression [31]. TLEO also restored redox balance: it normalized SOD and CAT enzyme activities and reduced oxidative markers (NO, ROS, MDA) in TNF‐α‐treated cells. This antioxidant capability contrasts with certain conventional anti‐inflammatory agents, whose mechanisms involve the suppression of endogenous antioxidant defenses and are associated with perturbations in redox homeostasis [32]. Green tea polyphenols, while effective antioxidants, require substantially higher concentrations (0.1%–1.0%) to achieve redox modulation in vitro, underscoring the potency of TLEO at low dose [33]. Additionally, WB analysis showed that TLEO significantly downregulated PTGS2 and MMP9 protein expression (*p* < 0.05; Figure 6), aligning with its predicted multi‐target mechanism. Restoration of EGFR expression emerged as a key reparative function of TLEO. Unlike conventional *Lavandula angustifolia* oils whose wound healing efficacy depends on compositional variability and lacks defined EGFR modulation, TLEO uniquely reinstated receptor levels to those of untreated controls pathways [31, 34] (Figure 6). This capacity for EGFR normalization is absent in antimicrobial‐focused oils such as *Melaleuca alternifolia*, which support skin integrity primarily through pathogen reduction rather than enhanced re‐epithelialization [35].

The distinct binding modes of linalool and linalyl acetate, observed in our double‐docking analysis, support the synergistic mechanism of TLEO. These compounds act through complementary pathways: linalyl acetate inhibits PTGS2 to reduce inflammation, while linalool stabilizes EGFR to enhance skin barrier repair. This dual targeting resembles findings in *Rosmarinus officinalis* oil, where carnosic acid and rosmarinic acid jointly suppress COX‐2 and MAPK pathways [36]. Notably, TLEO exhibits a multi‐target pharmacological profile that parallels the mechanism of established skin barrier modulators such as tacrolimus—particularly in the modulation of barrier lipids and ceramide metabolism [37]. In contrast, TLEO might have comparable multi‐modal benefits at a single low‐dose application, offering a favorable safety and compliance profile.

Safety considerations are critical for plant‐derived extracts, as essential oils exhibit variable composition and dose‐dependent toxicity. Rigorous toxicity assessments have demonstrated that high‐dose essential oil formulations can induce systemic adverse effects, underscoring the importance of minimizing effective concentration [29]. The robust efficacy of TLEO at 0.01% (v/v) positions it within a safe therapeutic window and supports its development as a topical dermatological agent.

To summarize, this study provides mechanistic evidence for TLEO as a multifunctional natural agent capable of addressing inflammation, oxidative stress, and barrier repair in a single low‐dose platform. By integrating systems biology with structural and cellular validation, these findings lay a foundation for advancing TLEO toward clinical translation and expand the paradigm of multi‐target natural products in dermatology.

## 5 Conclusions

TLEO exhibits considerable potential as a natural agent for the amelioration of inflammatory skin disorders, attributable to the synergistic interaction between linalool and linalyl acetate. These constituents mediate therapeutic effects through the suppression of inflammatory markers, attenuation of oxidative stress, and facilitation of skin barrier restoration. Preliminary evidence from in vitro and in vivo studies supports its incorporation into topical formulations designed to alleviate cutaneous irritation and improve skin health. These findings posit TLEO as a component of interest for the development of dermatological interventions. Further comprehensive studies are required to delineate its precise mechanisms and validate efficacy in human clinical trials.

However, some limitations should be noted. The use of public databases might miss important genes related to skin diseases. The HaCaT keratinocyte monoculture model lacks the multicellular complexity of human skin, including immune and fibroblast populations. Therefore, while this study provides mechanistic insight into epidermal responses, the findings may not fully represent the pathophysiology of skin diseases. Also, the study looked only at short‐term inflammation, while real skin diseases often involve long‐term changes. Docking studies predict how compounds bind to proteins but do not prove they actually block their function. More studies, such as animal tests, are needed to confirm these effects. Finally, absorption, distribution, metabolism, and excretion of TLEO in the body were not tested. Future work should focus on in vivo studies, human trials, and improved formulations to better deliver TLEO through the skin.

## Author Contributions

Liu Fei, Zhang Yingyu, and Minhazul Abedin designed the experiments. Zhang Yingyu drafted the manuscript. Zhang Yingyu and Minhazul Abedin conducted the investigation, methodology, and software analysis. Song Jingyi handled data curation; Yuan Lirong managed visualization. Wu Hua supervised the project, acquired funding, and edited the manuscript. Xiao Junsong and Wang Qian performed formal analysis. Yang Suzhen and Lou Hongxiang contributed to conceptualization and data curation. Dou Xixi assisted in investigation, and Li Qianqian in editing. All authors reviewed and approved the final manuscript.

## Funding

This research was supported by the Beijing Municipal Education Commission General Project (KM202010011010), the Shandong Postdoctoral Science Foundation (SDCX‐ZG‐202400156), and the Enterprise Technological Innovation Project of Shandong Province (202350100896).

## Conflicts of Interest

The authors declare no conflicts of interest.

## Supporting information


**Data S3:** Western Blot Validation of Key Signaling Proteins.

**Figure S1:** Gene Ontology (GO) analysis: Bar chart of biological process, cellular component, and molecular function categories.
**Figure S2:** Topological Networks, 3D Structural Models, and Core Component Formulations of TLEO. A‐C: Degree, betweenness and closeness centrality information on the topological networks of the top 12 targets. D: 3D structures of 9 key predicted protein targets. E: Structural formulas of 12 core components of TLEO.
**Figure S3:** The 2D and 3D interactions of TLEO active components with MMP9.
**Figure S4:** The 2D and 3D interactions of TLEO active components with EGFR.
**Figure S5:** The 2D and 3D interactions of TLEO active components with PTGS2.
**Figure S6:** Construction of TNFα stimulated HaCaT cell model. A: The effect of different concentrations of TNF‐α and TLEO on the activity of HaCaT cells. B‐D: The effect of different concentrations of TNF alpha on the expression of IL‐6, IL‐1β, and IL‐8.

**Table S1:** ADMET analysis of TLEO components.
**Table S2:** Network pharmacology analysis of core component and core target.
**Table S3:** Gene name, uniprot ID and its subcellular localization of core target.
**Table S4:** Annotation of KEGG pathways with enrichment degree.
**Table S5:** Docking score of the core TLEO components and core targets.

## Data Availability

The data that support the findings of this study are available from the corresponding author upon reasonable request.
