# Supplementary material for: Unveiling the Therapeutic Potential of “Taikong Blue” Lavender Essential Oil and Its Key Compounds in Skin Problems via Network Pharmacology and In Vitro Validation
Source: J Cosmet Dermatol. 2026 Jan 28;25(2):e70640. doi: 10.1111/jocd.70640 (PMC12848645; doi:10.1111/jocd.70640)
Supplement: Supplementary file 1 — Data S3: Western Blot Validation of Key Signaling Proteins. [file JOCD-25-e70640-s003.pdf]

Supplementary Data 3: Western Blot Validation of Key Signaling Proteins

This supplementary data presents Western Blot validation of key target proteins predicted by network pharmacology in TNF-α-induced inflammatory model.

- **Target Proteins: Page 1: EGFR; Page 2: PTGS2 (COX-2) ; Page 3: MMP9 ; Pages 4-5: p38 MAPK and p-p38 MAPK ; Pages 6-7: NF-κB p65 and p-NF-κB p65**

Treatment Groups: Control, TNF-α, TNF-α + TLEO, TNF-α + linalool, TNF-α + linalyl acetate

Image Layout:

- **Upper left: Cropped bands for quantitative analysis (6 bands, n=3 biological replicates)**
- **Lower left: Original images of target protein bands with molecular weight markers**
- **Right: Complete unedited original membrane images including target proteins and loading controls (β-actin or GAPDH)**

This data provides complete evidence chain from original imaging to analysis, validating the key signaling pathways (EGFR, MAPK, NF-κB) predicted by network pharmacology.

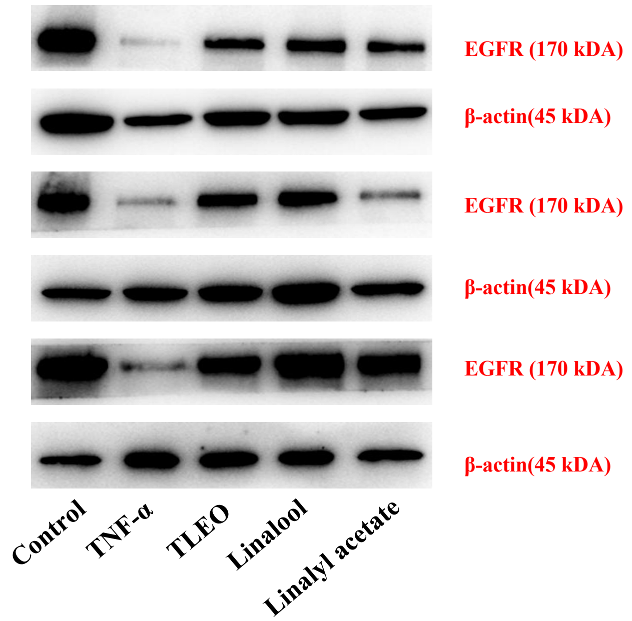

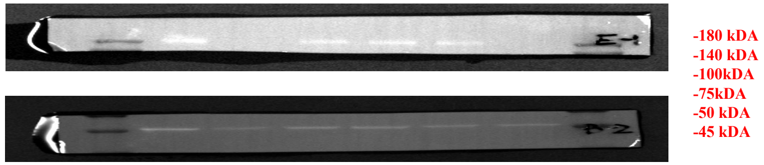

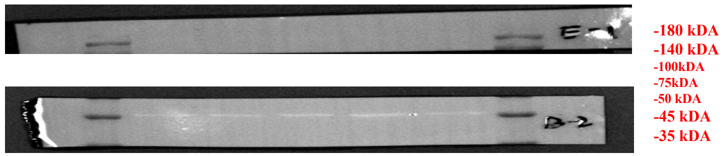

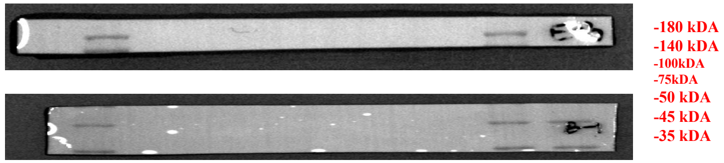

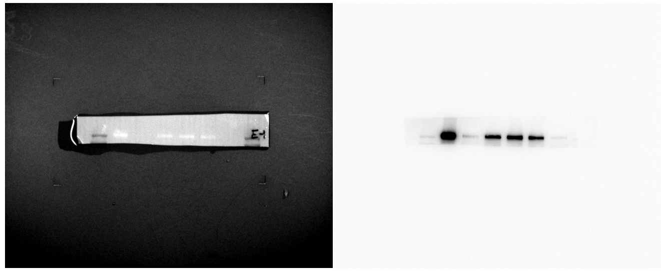

EGFR（170 kDA）

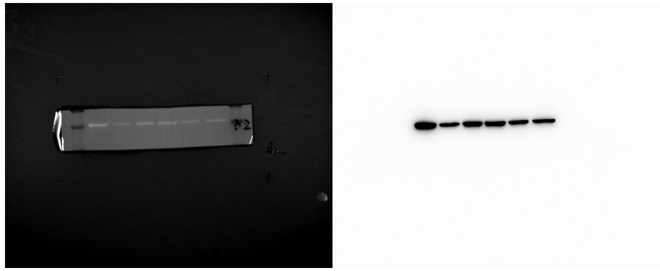

β-actin（45 kDA）

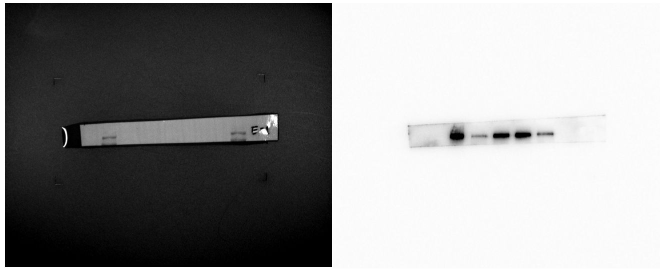

EGFR（170 kDA）

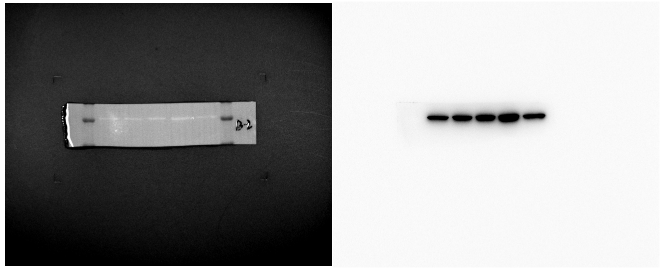

β-actin（45 kDA）

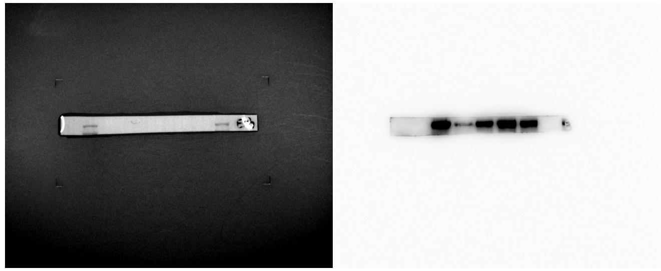

EGFR（170 kDA）

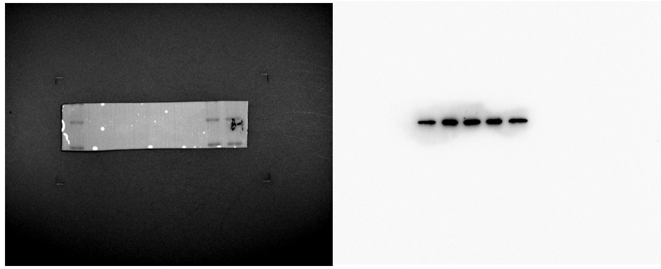

β-actin（45 kDA）

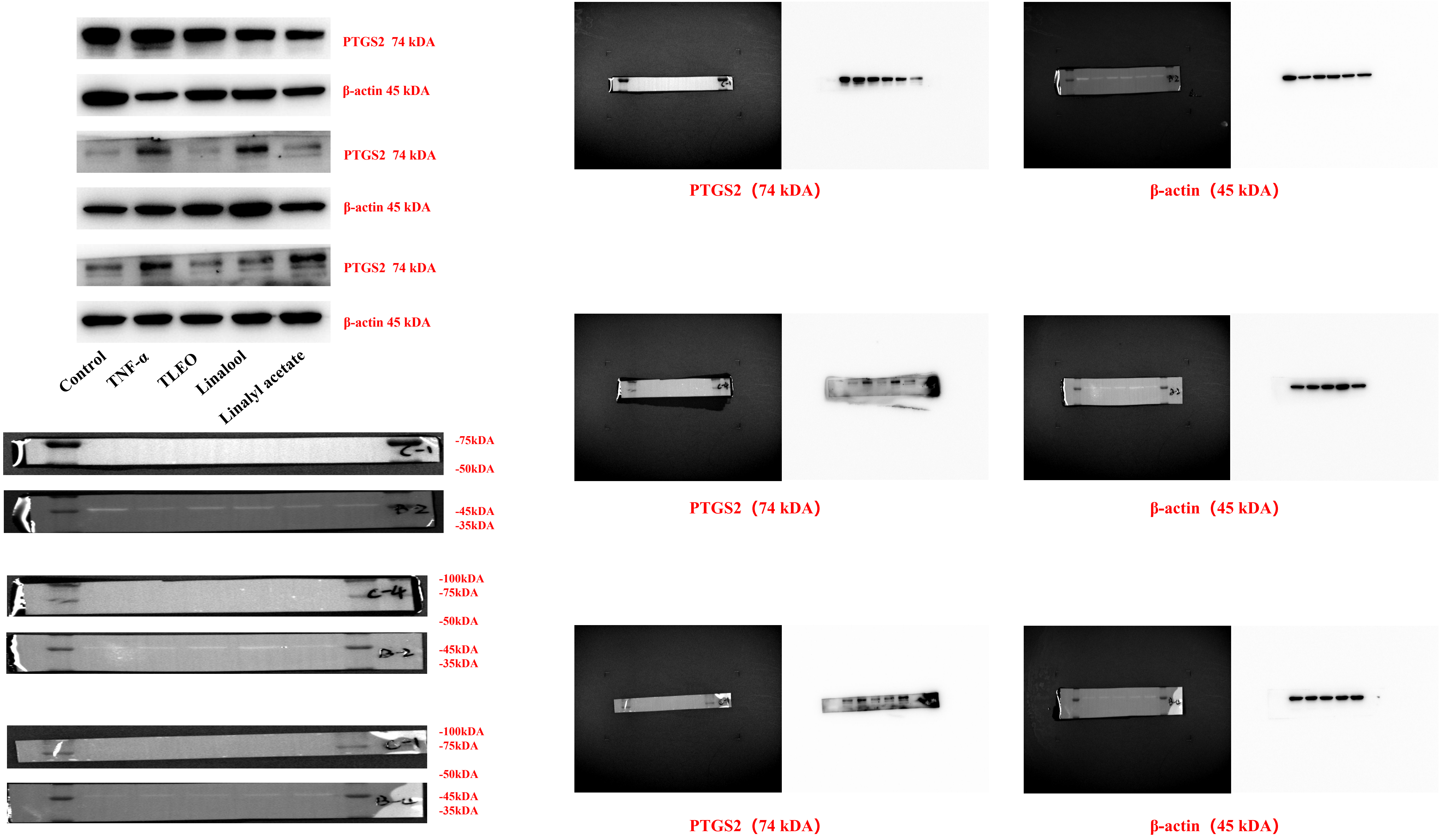

PTGS2 74 kDA
β-actin 45 kDA
PTGS2 74 kDA
β-actin 45 kDA
PTGS2 74 kDA
β-actin 45 kDA
Control
TNF-α
TLEO
Linalool
Linalyl acetate
-75kDA
-50kDA
-45kDA
-35kDA
-100kDA
-75kDA
-50kDA
-45kDA
-35kDA
-100kDA
-75kDA
-50kDA
-45kDA
-35kDA
PTGS2（74 kDA）
β-actin（45 kDA）
PTGS2（74 kDA）
β-actin（45 kDA）
PTGS2（74 kDA）
β-actin（45 kDA）

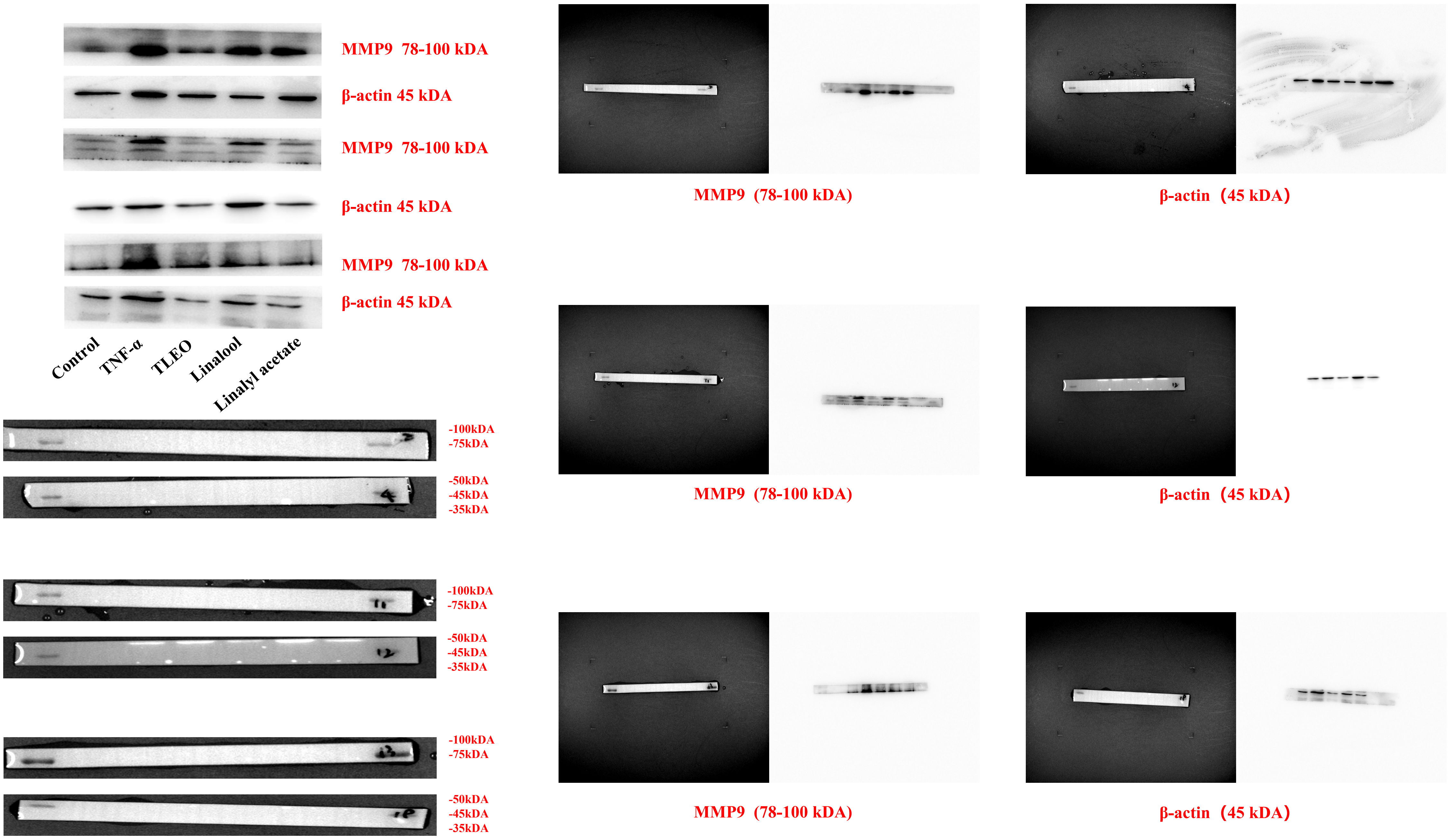

MMP9 78-100 kDA
β-actin 45 kDA
MMP9 78-100 kDA
β-actin 45 kDA
MMP9 78-100 kDA
β-actin 45 kDA
Control
TNF-α
TLEO
Linalool
Linalyl acetate
-100kDA
-75kDA
-50kDA
-45kDA
-35kDA
-100kDA
-75kDA
-50kDA
-45kDA
-35kDA
-100kDA
-75kDA
-50kDA
-45kDA
-35kDA
MMP9 (78-100 kDA)
β-actin (45 kDA)
MMP9 (78-100 kDA)
β-actin (45 kDA)
MMP9 (78-100 kDA)
β-actin (45 kDA)

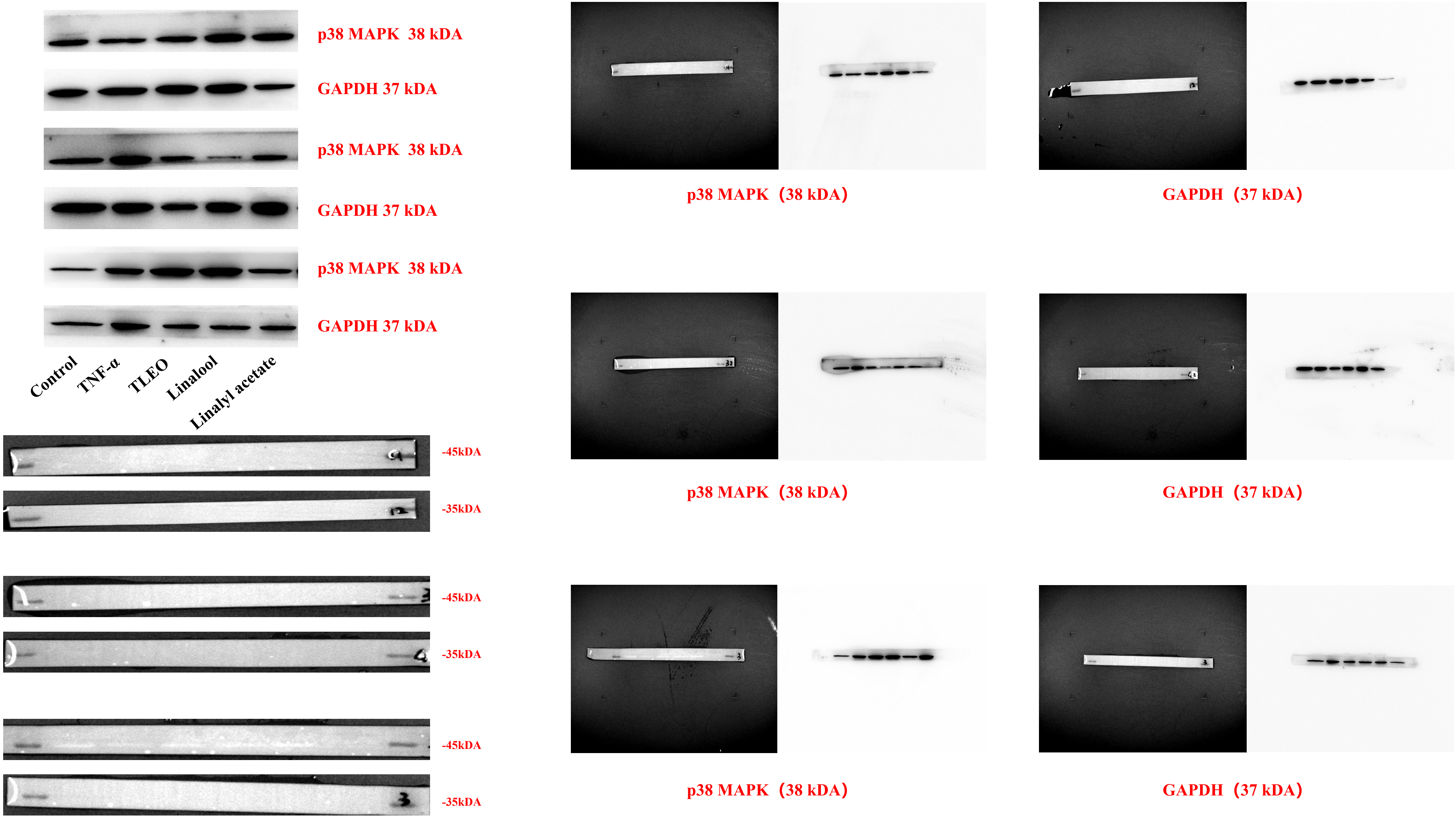
p38 MAPK 38 kDA
GAPDH 37 kDA
p38 MAPK 38 kDA
GAPDH 37 kDA
p38 MAPK 38 kDA
GAPDH 37 kDA
Control
TNF-α
TLEO
Linalool
Linalyl acetate
-45kDA
-35kDA
-45kDA
-35kDA
-45kDA
-35kDA
p38 MAPK（38 kDA）
GAPDH（37 kDA）
p38 MAPK（38 kDA）
GAPDH（37 kDA）
p38 MAPK（38 kDA）
GAPDH（37 kDA）

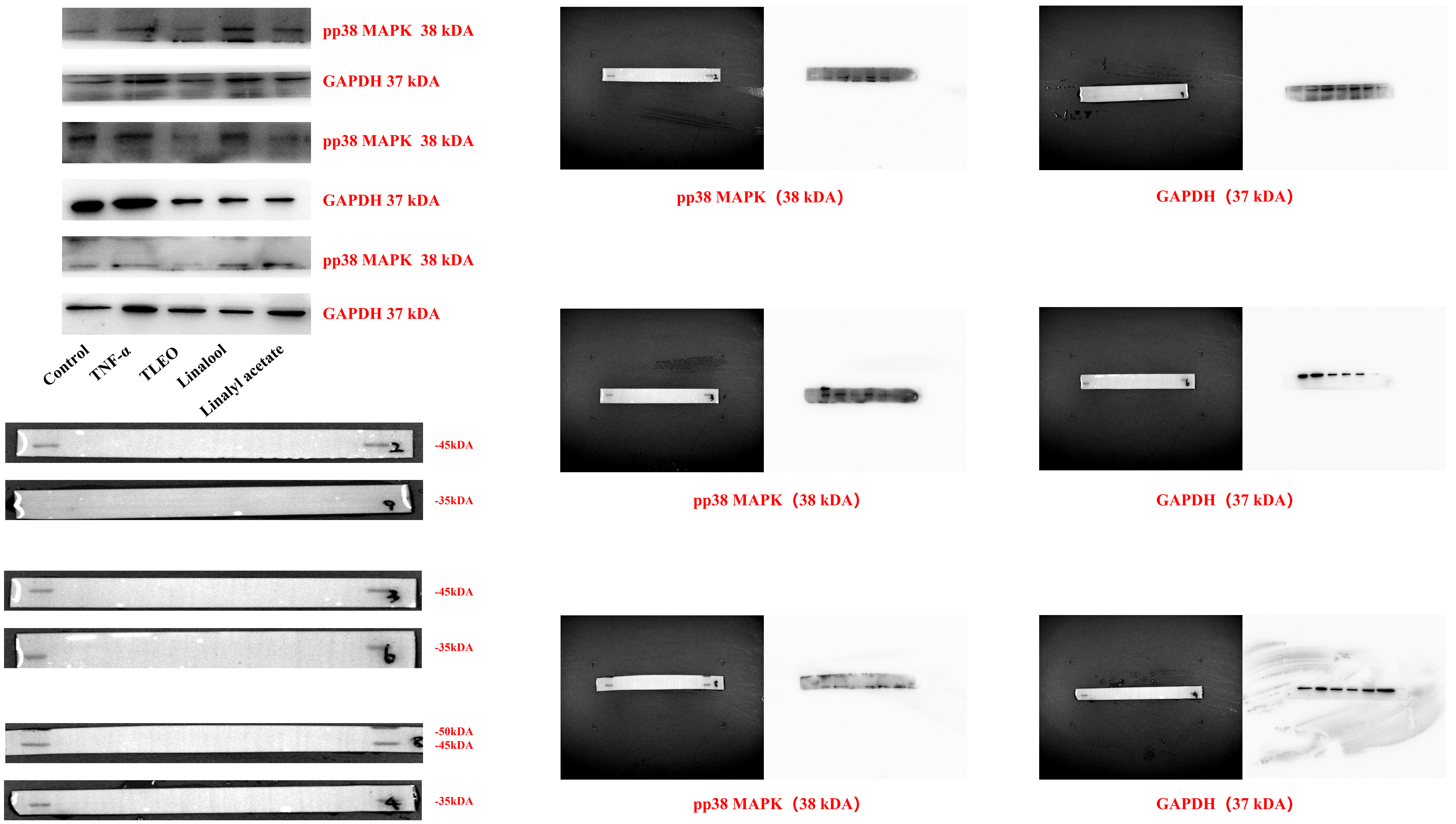
pp38 MAPK 38 kDA
GAPDH 37 kDA
pp38 MAPK 38 kDA
GAPDH 37 kDA
pp38 MAPK 38 kDA
GAPDH 37 kDA
Control
TNF-α
TLEO
Linalool
Linalyl acetate
-45kDA
-35kDA
-45kDA
-35kDA
-50kDA
-45kDA
-35kDA
pp38 MAPK（38 kDA）
GAPDH（37 kDA）
pp38 MAPK（38 kDA）
GAPDH（37 kDA）
pp38 MAPK（38 kDA）
GAPDH（37 kDA）

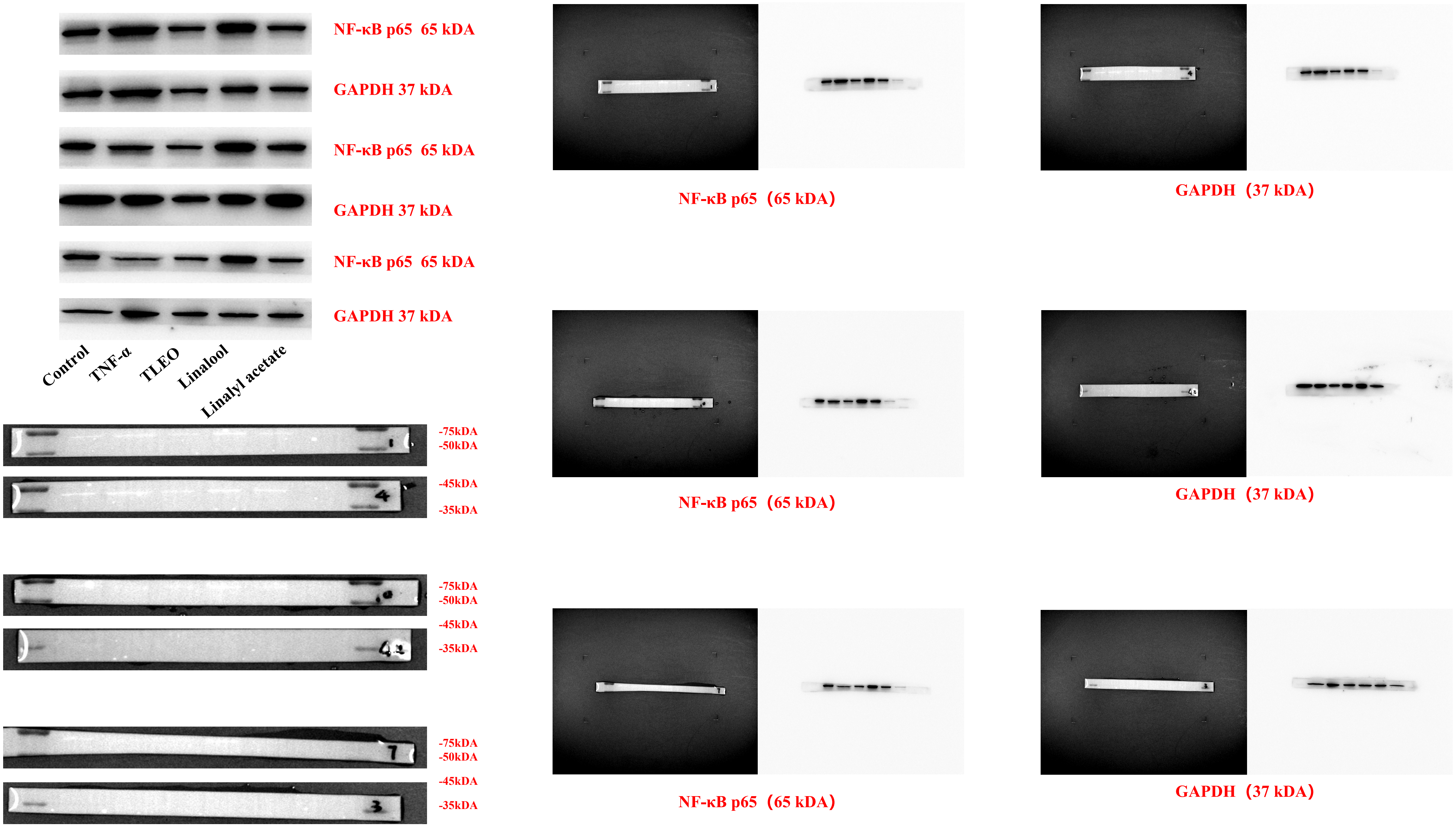
NF-κB p65 65 kDA
GAPDH 37 kDA
NF-κB p65 65 kDA
GAPDH 37 kDA
NF-κB p65 65 kDA
GAPDH 37 kDA
Control
TNF-α
TLEO
Linalool
Linalyl acetate
-75kDA
-50kDA
-45kDA
-35kDA
-75kDA
-50kDA
-45kDA
-35kDA
-75kDA
-50kDA
-45kDA
-35kDA
NF-κB p65 (65 kDA)
GAPDH (37 kDA)
NF-κB p65 (65 kDA)
GAPDH (37 kDA)
NF-κB p65 (65 kDA)
GAPDH (37 kDA)

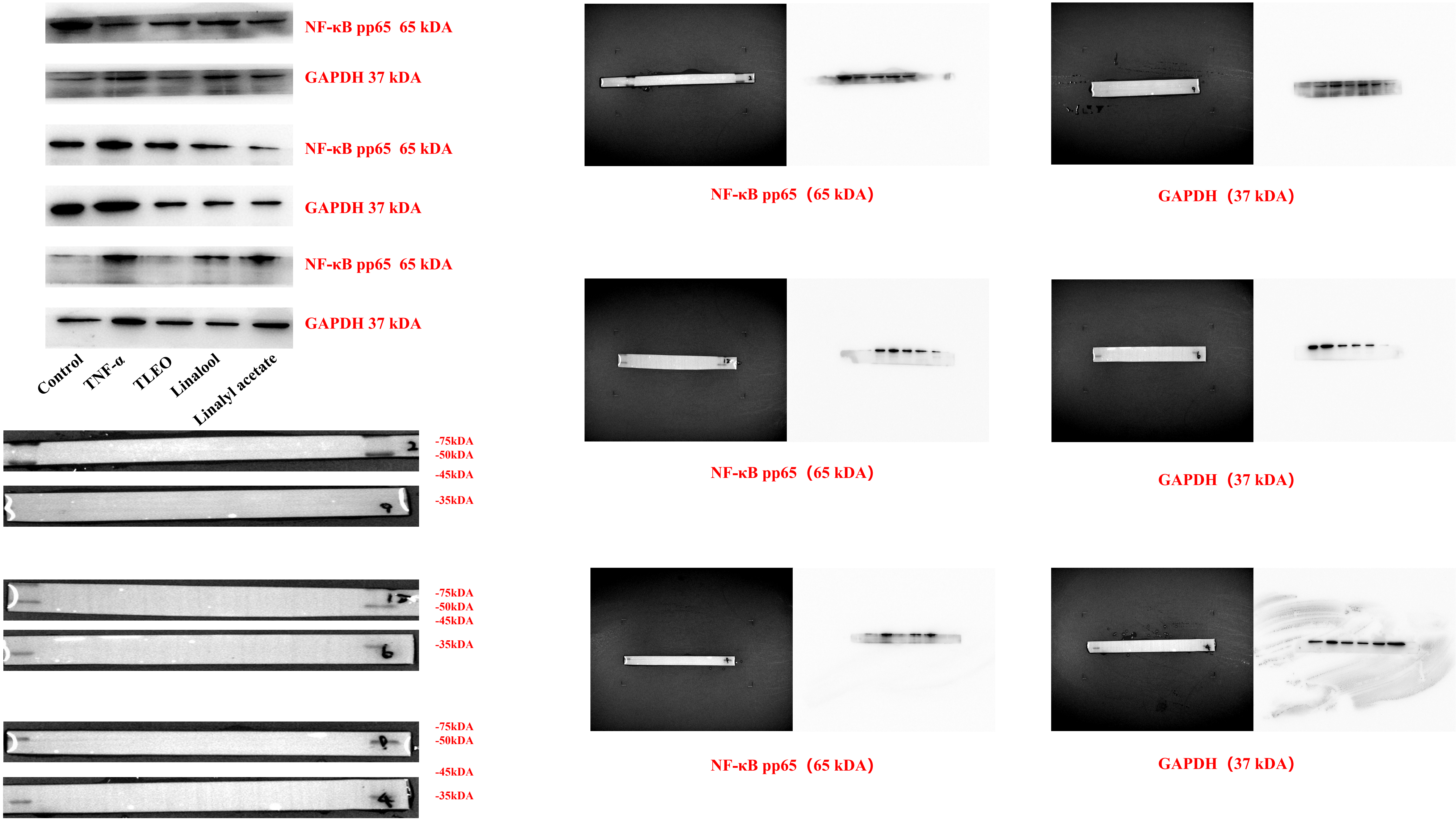

NF-κB pp65 65 kDA
GAPDH 37 kDA
NF-κB pp65 65 kDA
GAPDH 37 kDA
NF-κB pp65 65 kDA
GAPDH 37 kDA
Control
TNF-α
TLEO
Linalool
Linalyl acetate
-75kDA
-50kDA
-45kDA
-35kDA
-75kDA
-50kDA
-45kDA
-35kDA
-75kDA
-50kDA
-45kDA
-35kDA
NF-κB pp65（65 kDA）
GAPDH（37 kDA）
NF-κB pp65（65 kDA）
GAPDH（37 kDA）
NF-κB pp65（65 kDA）
GAPDH（37 kDA）
