# Supplementary material for: Unveiling the Therapeutic Potential of “Taikong Blue” Lavender Essential Oil and Its Key Compounds in Skin Problems via Network Pharmacology and In Vitro Validation
Source: J Cosmet Dermatol. 2026 Jan 28;25(2):e70640. doi: 10.1111/jocd.70640 (PMC12848645; doi:10.1111/jocd.70640)
Supplement: Supplementary file 2 — Figure S1: Gene Ontology (GO) analysis: Bar chart of biological process, cellular component, and molecular function categories. Figure S2: Topological Networks, 3D Structural Models, and Core Component Formulations of TLEO. A‐C: Degree, betweenness and closeness centrality information on the topological networks of the top 12 targets. D: 3D structures of 9 key predicted protein targets. E: Structural formulas of 12 core components of TLEO. Figure S3: The 2D and 3D interactions of TLEO active components with MMP9. Figure S4: The 2D and 3D interactions of TLEO active components with EGFR. Figure S5: The 2D and 3D interactions of TLEO active components with PTGS2. Figure S6: Construction of TNFα stimulated HaCaT cell model. A: The effect of different concentrations of TNF‐α and TLEO on the activity of HaCaT cells. B‐D: The effect of different concentrations of TNF alpha on the expression of IL‐6, IL‐1β, and IL‐8. [file JOCD-25-e70640-s002.docx]

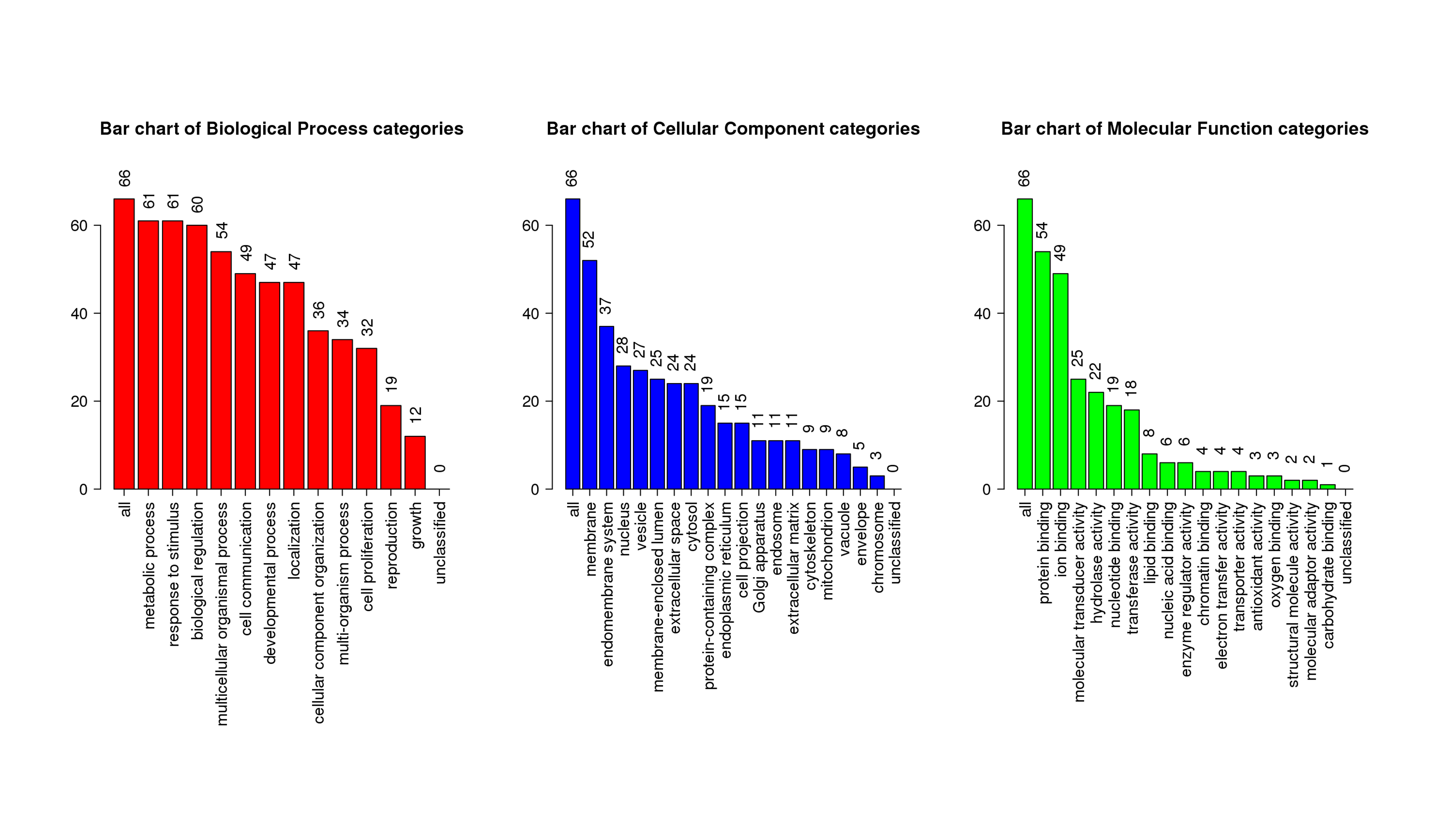


**Fig.S1: Gene Ontology (GO) analysis: Bar chart of biological process, cellular component, and molecular function categories.**


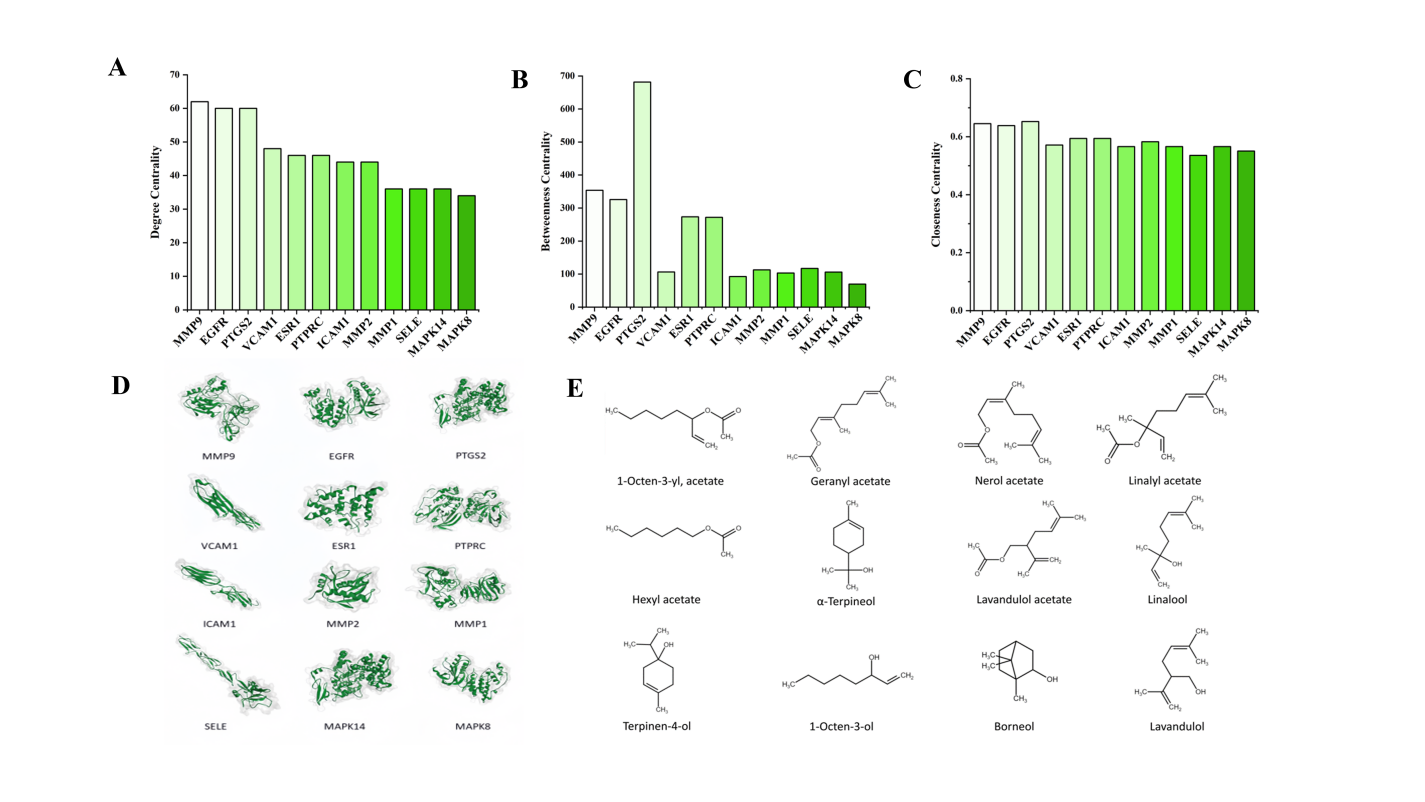


**Fig.S2: Topological Networks, 3D Structural Models, and Core Component Formulations of TLEO. A-C: Degree, betweenness and closeness centrality information on the topological networks of the top 12 targets. D: 3D structures of 9 key predicted protein targets. E: Structural formulas of 12 core components of TLEO.**


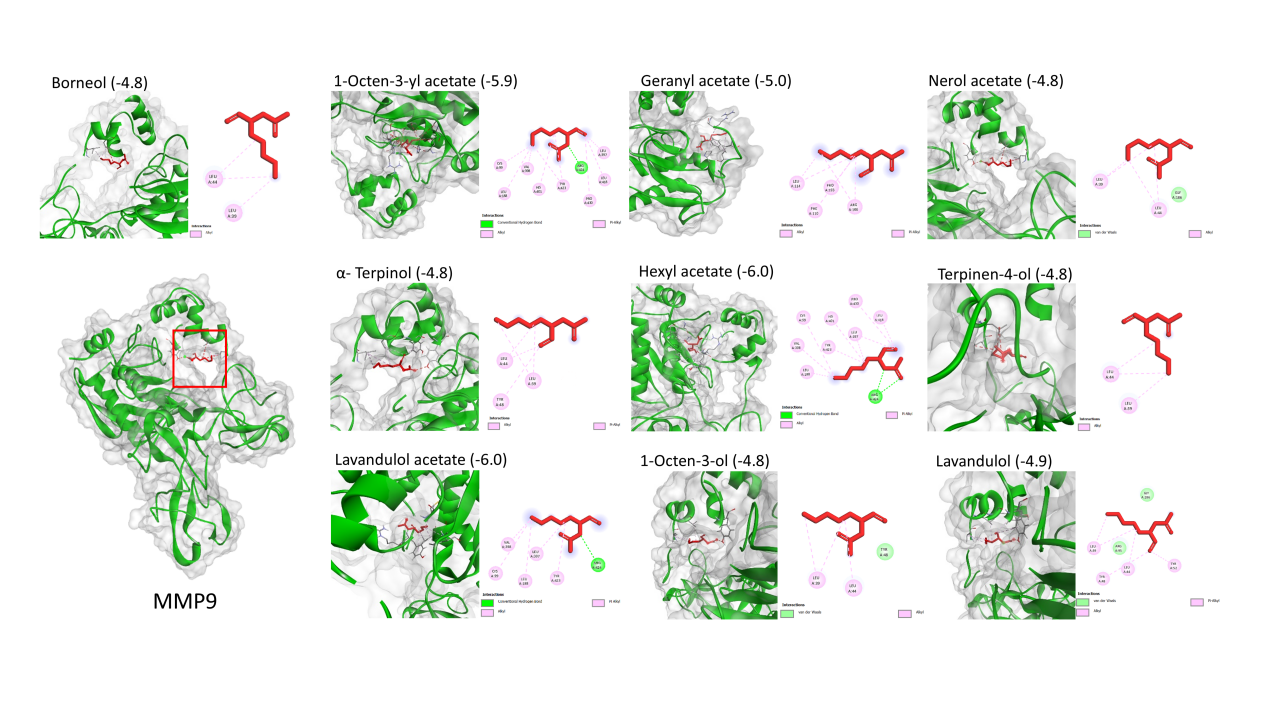


**Fig.S3: The 2D and 3D interactions of TLEO active components with MMP9.**


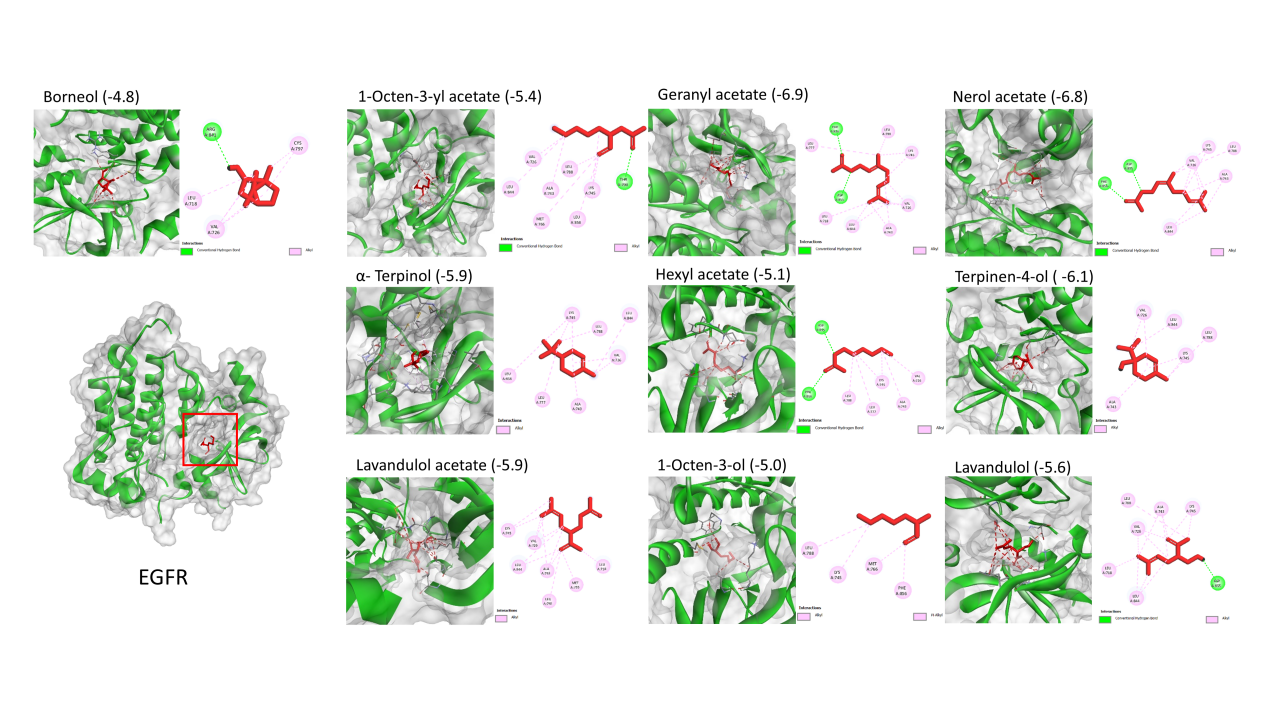


**Fig.S4: The 2D and 3D interactions of TLEO active components with EGFR.**


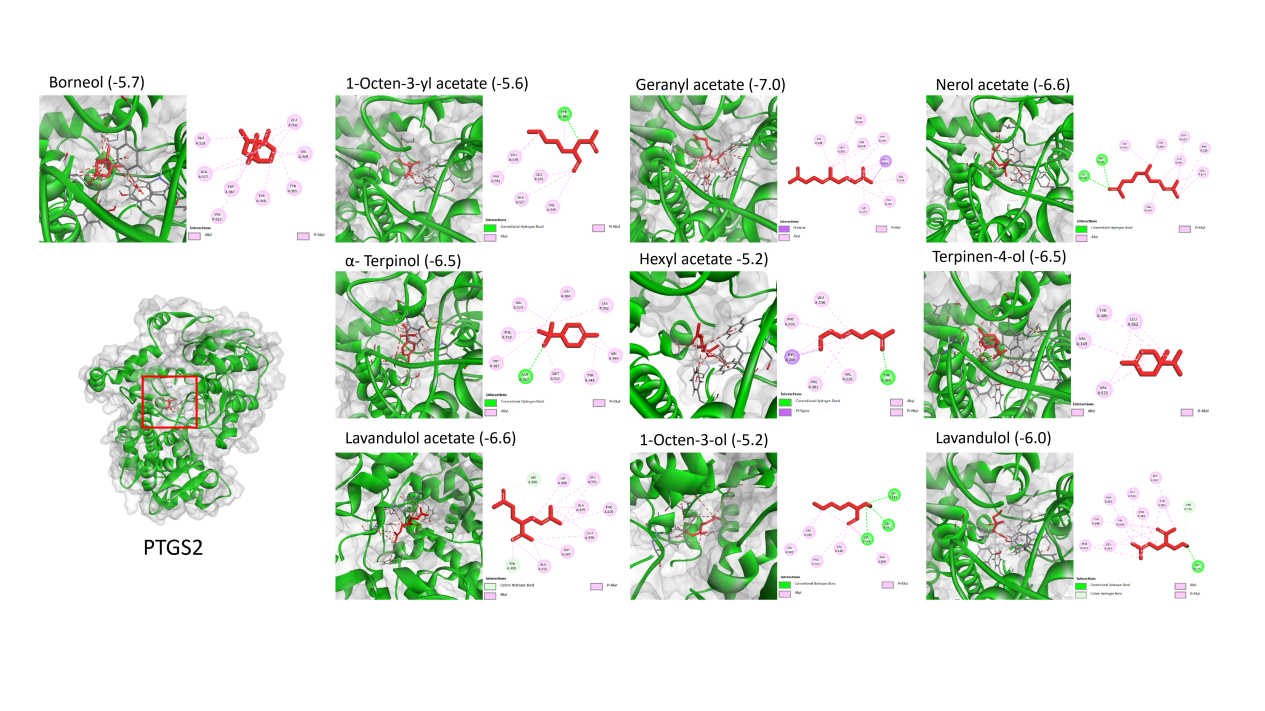


**Fig.S5: The 2D and 3D interactions of TLEO active components with PTGS2.**

**
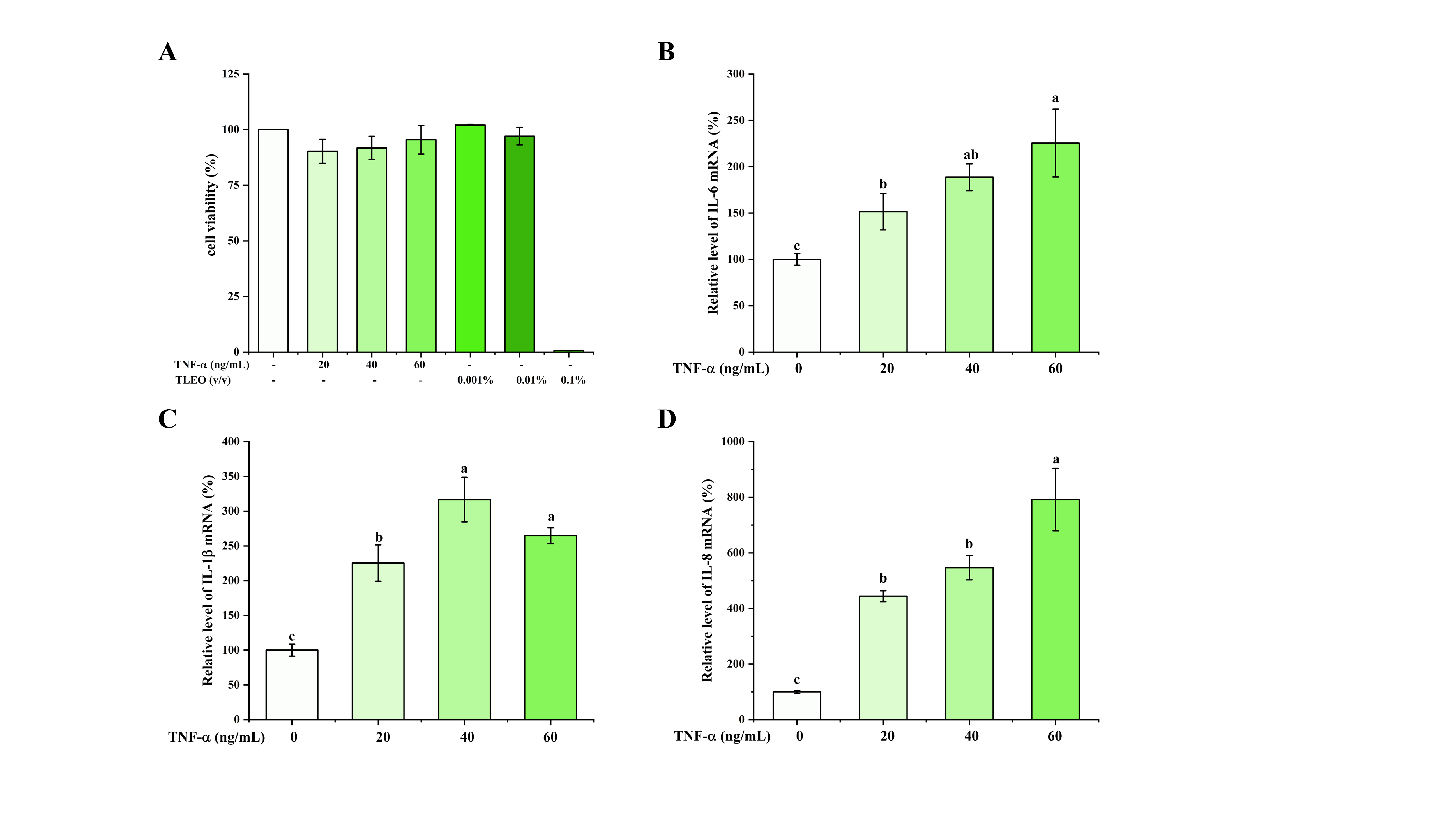
**

**Fig.S6: Construction of TNFα stimulated HaCaT cell model. A: The effect of different concentrations of TNF-α and TLEO on the activity of HaCaT cells. B-D: The effect of different concentrations of TNF alpha on the expression of IL-6, IL-1β, and IL-8.**
