# Supplementary material for: Unveiling the Therapeutic Potential of “Taikong Blue” Lavender Essential Oil and Its Key Compounds in Skin Problems via Network Pharmacology and In Vitro Validation
Source: J Cosmet Dermatol. 2026 Jan 28;25(2):e70640. doi: 10.1111/jocd.70640 (PMC12848645; doi:10.1111/jocd.70640)
Supplement: Supplementary file 3 — Table S1: ADMET analysis of TLEO components. Table S2: Network pharmacology analysis of core component and core target. Table S3: Gene name, uniprot ID and its subcellular localization of core target. Table S4: Annotation of KEGG pathways with enrichment degree. Table S5: Docking score of the core TLEO components and core targets. [file JOCD-25-e70640-s001.docx]

**Table S1:** ADMET analysis of TLEO components

| **Compound Name** | **Average Percentage** | **Pubchem ID** | **M.W** | **OB%** | **CaCO-2** | **DL** | **Log Kp (skin permeation** | **GI absorption** | **BBB permeant** |
| --- | --- | --- | --- | --- | --- | --- | --- | --- | --- |
| α-Pinene | 0.092 | 440968 | 136.26 | 46.25 | 1.85 | 0.05 | -3.95 cm/s | High | yes |
| 2-Thujene | 0.185 | 520384 | 136.26 | 46.44 | 1.82 | 0.04 | -4.71 cm/s | Low | yes |
| Camphene | 0.044 | 92221 | 136.26 | 34.98 | 1.81 | 0.04 | -4.13 cm/s | Low | yes |
| Butyl acetate | 0.067 | 31272 | 116.18 | 42.65 | 1.13 | 0.01 | -5.74 cm/s | High | yes |
| β-Pinene | 0.027 | 10290825 | 136.26 | 44.77 | 1.85 | 0.05 | -4.18 cm/s | Low | yes |
| Sabinen | 0.025 | 18818 | 136.26 | 45.15 | 1.8 | 0.04 | -4.94 cm/s | Low | yes |
| 3-Carene | 0.033 | 443156 | 136.26 | 45.2 | 1.84 | 0.04 | -4.02 cm/s | Low | yes |
| β-Myrcene | 1.152 | 31253 | 136.26 | 24.96 | 1.84 | 0.04 | -4.17 cm/s | Low | yes |
| α-Terpine | 0.05 | 7462 | 136.26 | 33.95 | 1.84 | 0.02 | -4.11 cm/s | Low | yes |
| D-Limonene | 0.313 | 440917 | 136.26 | 39.84 | 1.83 | 0.05 | -3.89 cm/s | Low | yes |
| Eucalyptol | 0.697 | 2758 | 154.28 | 39.73 | 1.57 | 0.05 | -5.30 cm/s | High | yes |
| Butyl butylate | 0.177 | 7983 | 144.24 | 40.73 | 1.25 | 0.02 | -5.63 cm/s | High | yes |
| trans-beta-Ocimene | 2.558 | 5281553 | 136.23 | 15.06 | 1.85 | 0.05 | -4.11 cm/s | Low | yes |
| cis-β-Ocimene | 0.825 | 18756 | 136.26 | 15.06 | 1.85 | 0.04 | -4.11 cm/s | Low | yes |
| Hexyl acetate | 0.667 | 8908 | 144.24 | 13.28 | 1.21 | 0.04 | -5.46 cm/s | High | yes |
| Terpinolene | 0.078 | 11463 | 136.26 | 29.62 | 1.86 | 0.02 | -3.96 cm/s | Low | yes |
| Hexyl isobutyrate | 0.07 | 16872 | 172.26 |  |  |  | -4.89 cm/s | High | yes |
| Hexyl formate | 0.023 | 61177 | 130.21 | 19.17 | 1.13 | 0.01 | -5.35 cm/s | High | yes |
| (4E,6E)-Alloocimene | 0.812 | 5368821 | 136.26 | 14.89 | 1.85 | 0.05 | -4.15 cm/s | Low | yes |
| 1-Octen-3-yl, acetate | 2.194 | 17121 | 170.25 | 42.33 | 1.43 | 0.04 | -5.12 cm/s | High | yes |
| Butyl caproate | 0.024 | 12294 | 172.26 | 18.93 | 1.22 | 0.03 | -5.04 cm/s | High | yes |
| n-Hexyl butanoate | 0.389 | 17525 | 172.3 | 19.58 | 1.19 | 0.03 | -5.04 cm/s | High | yes |
| cis-Linalool oxide | 0.075 | 6428573 | 170.25 | 53.37 | 0.92 | 0.03 | -5.61 cm/s | High | yes |
| Matsutake alcohol | 0.351 | 18827 | 128.21 | 40.11 | 1.19 | 0.01 | -5.27 cm/s | High | no |
| trans-Sabinene hydrate | 0.082 | 12315151 | 154.28 | 65.09 | 1.27 | 0.05 | -5.74 cm/s | High | yes |
| trans-Linalool oxide | 0.077 | 6432254 | 170.25 | 22.91 | 0.99 | 0.07 | -5.61 cm/s | High | yes |
| 1-Octen-3-ol | 0.43 | 18827 | 128.21 | 31.33 | 1.25 | 0.5 | -5.64 | High | yes |
| Copaene | 0.038 | 12303902 | 204.35 | 24.08 | 1.81 | 0.12 | -4.37 cm/s | Low | yes |
| Bornanone | 0.121 | 159055 | 152.26 | 67.17 | 1.29 | 0.05 | -5.67 cm/s | High | yes |
| Linalool | 32.355 | 6549 | 154.25 | 58.18 | 1.29 | 0.04 | -5.13 cm/s | High | yes |
| Linalyl acetate | 42.438 | 6999980 | 196.32 | 36.84 | 1.4 | 0.04 | -4.71 cm/s | High | yes |
| α-Santalene | 0.122 | 12315252 | 204.39 | 17.17 | 1.86 | 0.11 | -3.85 cm/s | Low | no |
| Bornyl acetate | 0.081 | 6950274 | 196.32 | 65.55 | 1.3 | 0.08 | -4.44 cm/s | High | yes |
| Caryophyllene | 2.2 | 6429301 | 204.39 | 30.29 | 1.82 | 0.05 | -4.44 cm/s | Low | yes |
| Terpinen-4-ol | 4.038 | 2724161 | 154.28 | 81.41 | 1.36 | 0.04 | -4.93 cm/s | High | yes |
| Lavandulol acetate | 2.798 | 30247 | 196.29 | 45 | 1.32 | 0.4 | -4.94 cm/s | High | yes |
| cis-β-Farnesene | 0.025 | 5317319 | 204.39 | 6.75 | 1.92 | 0.05 | -3.27 cm/s | Low | no |
| trans-β-Farnesene | 1.166 | 5281517 | 204.39 | 17.42 | 1.95 | 0.05 | -3.27 cm/s | Low | yes |
| Humulene | 0.084 | 5281520 | 204.39 | 22.98 | 1.88 | 0.06 | -4.32 cm/s | Low | no |
| Lavandulol | 0.222 | 5464156 | 154.28 | 38.55 | 1.25 | 0.04 | -5.10 cm/s | High | yes |
| Cryptone | 0.09 | 642520 | 138.23 | 49.91 | 1.31 | 0.02 | -5.71 cm/s | High | yes |
| α-Terpineol | 0.292 | 17100 | 152.31 | 29.14 | 1.82 | 0.04 | -4.83 cm/s | High | yes |
| Borneol | 0.375 | 6552009 | 154.28 | 81.8 | 1.22 | 0.05 | -5.31 cm/s | High | yes |
| D-Germacrene | 0.279 | 5317570 | 204.39 | 19.22 | 1.83 | 0.06 | -4.18 cm/s | High | yes |
| Nerol acetate | 0.4 | 1549025 | 196.32 | 57.47 | 1.25 | 0.04 | -4.63 cm/s | High | yes |
| Carvone | 0.031 | 16724 | 150.24 | 47.43 | 1.34 | 0.05 | -5.29 cm/s | High | yes |
| Geranyl acetate | 0.862 | 1549026 | 196.32 | 25.94 | 1.28 | 0.04 | -4.63 cm/s | High | yes |
| Cuminal | 0.055 | 326 | 148.22 | 38.29 | 1.39 | 0.03 | -5.52 cm/s | High | yes |
| Nerol | 0.066 | 643820 | 154.28 | 35.66 | 1.15 | 0.02 | -4.71 cm/s | High | yes |
| Guaniol | 0.189 | 637566 | 154.28 | 23.93 | 1.19 | 0.02 | -4.71 cm/s | High | yes |
| p-Cymen-8-ol | 0.023 | 14529 | 150.24 | 32.26 | 1.33 | 0.03 | -5.80 cm/s | High | yes |
| 3,7-Octadiene-2,6-diol | 0.029 | 637566 | 154.28 | 23.93 | 1.19 | 0.02 | -4.71 cm/s | High | yes |
| Caryophyllene oxide | 0.087 | 1742210 | 235.38 | 45.75 | 1.09 | 0.15 | -5.12 cm/s | High | yes |
| Cuminol | 0.029 | 325 | 150.24 | 42.57 | 1.2 | 0.03 | -5.55 cm/s | High | yes |
| T-Cadinol | 0.165 | 160799 | 222.41 | 28.59 | 1.41 | 0.09 | -5.29 cm/s | High | yes |
| Coumarin | 0.051 | 323 | 146.15 | 29.17 | 1.2 | 0.04 | -6.20 cm/s | High | yes |

**Table S2:** Network pharmacology analysis of core component and core target

| **Classification** | **No** | **Name** | **Degree** | **Betweenness** | **Closeness** |
| --- | --- | --- | --- | --- | --- |
| **Core Components** | 1 | 1-Octen-3-yl acetate | 104 | 20171.36 | 0.4752 |
|  | 2 | Geranyl acetate | 104 | 13518.719 | 0.4744409 |
|  | 3 | Nerol acetate | 104 | 14027.462 | 0.4744409 |
|  | 4 | Linalyl acetate | 77 | 12388.821 | 0.43740794 |
|  | 5 | α-Terpineol | 51 | 9858.181 | 0.4008097 |
|  | 6 | Hexyl acetate | 47 | 7107.226 | 0.40189445 |
|  | 7 | Lavandulol acetate | 46 | 9966.498 | 0.3965287 |
|  | 8 | Linalool | 38 | 4574.9097 | 0.39078948 |
|  | 9 | Terpinen-4-ol | 37 | 4401.0312 | 0.38372093 |
|  | 10 | 1-Octen-3-ol | 33 | 3487.9854 | 0.3802817 |
|  | 11 | Borneol | 30 | 5064.3545 | 0.37264743 |
|  | 12 | Lavandulol | 23 | 2669.2786 | 0.3689441 |
| **Core Targets** | 1 | MMP9 | 62 | 353.89273 | 0.6451613 |
|  | 2 | EGFR | 60 | 325.56396 | 0.63829786 |
|  | 3 | PTGS2 | 60 | 681.5648 | 0.65217394 |
|  | 4 | VCAM1 | 48 | 106.28843 | 0.5714286 |
|  | 5 | ESR1 | 46 | 273.3516 | 0.5940594 |
|  | 6 | PTPRC | 46 | 271.8101 | 0.5940594 |
|  | 7 | ICAM1 | 44 | 92.360115 | 0.5660377 |
|  | 8 | MMP2 | 44 | 112.77355 | 0.5825243 |
|  | 9 | MMP1 | 36 | 103.119026 | 0.5660377 |
|  | 10 | SELE | 36 | 116.304855 | 0.53571427 |
|  | 11 | MAPK14 | 36 | 105.93504 | 0.5660377 |
|  | 12 | MAPK8 | 34 | 69.678375 | 0.5504587 |

**Table S3:** Gene name, uniprot ID and its subcellular localization of core target

| **Uniprot ID** | **Gene Name** | **Protein Name** | **Subcellular Localization** |
| --- | --- | --- | --- |
| P14780 | MMP9 | Matrix metallopeptidase 9 | Cytosol |
| P00533 | EGFR | Epidermal growth factor receptor | Plasma Membrane |
| P35354 | PTGS2 | ProstaglandinG/H synthase 2 | Cytoplasm |
| P19320 | VCAM1 | Vascular cell adhesion protein1 | Plasma Membrane |
| P03372 | ESR1 | Estrogen receptor1 | Vesicles |
| P08575 | PTPRC | Receptor-typetyrosine-proteinphosphatase C | Plasma Membrane |
| P05362 | ICAM1 | Intercellular adhesion molecule 1 | Plasma Membrane |
| P08253 | MMP2 | Matrix metallopeptidase 2 | Cytosol |
| P03956 | MMP1 | Matrix metallopeptidase 1 | Cytosol |
| P16581 | SELE | E-selectin | Plasma Membrane |
| Q16539 | MAPK14 | Mitogen-activated protein kinase 14 | Cytosol |
| P45983 | MAPK8 | Mitogen-activated protein kinase 8 | Cytosol |

**Table S4:** Annotation of KEGG pathways with enrichment degree.

| **Gene Set** | **Description** | **Size** | **Expect** | **Ratio** | **P Value** | **FDR** |
| --- | --- | --- | --- | --- | --- | --- |
| hsa05418 | Fluid shear stress and atherosclerosis | 13 | 1.2097 | 10.747 | 1.31E-10 | 4.28E-08 |
| hsa04668 | TNF signaling pathway | 11 | 0.95729 | 11.491 | 1.96E-09 | 3.20E-07 |
| hsa05200 | Pathways in cancer | 20 | 4.5776 | 4.3691 | 8.98E-09 | 9.76E-07 |
| hsa05167 | Kaposi sarcoma-associated herpesvirus infection | 12 | 1.6187 | 7.4134 | 5.08E-08 | 0.000004137 |
| hsa05120 | Epithelial cell signaling in Helicobacter pylori infection | 8 | 0.59178 | 13.519 | 1.04E-07 | 6.7514E-06 |
| hsa01522 | Endocrine resistance | 9 | 0.85286 | 10.553 | 1.39E-07 | 0.000007526 |
| hsa05163 | Human cytomegalovirus infection | 12 | 1.9581 | 6.1284 | 4.09E-07 | 0.000019063 |
| hsa04062 | Chemokine signaling pathway | 11 | 1.6448 | 6.6877 | 5.54E-07 | 0.000022563 |
| hsa05205 | Proteoglycans in cancer | 11 | 1.7492 | 6.2885 | 1.0233E-06 | 0.000037067 |
| hsa04657 | IL-17 signaling pathway | 8 | 0.80935 | 9.8845 | 1.2027E-06 | 0.000039209 |
| hsa04621 | NOD-like receptor signaling pathway | 10 | 1.462 | 6.8397 | 1.5616E-06 | 0.000045117 |
| hsa05215 | Prostate cancer | 8 | 0.84416 | 9.4769 | 1.6607E-06 | 0.000045117 |
| hsa04933 | AGE-RAGE signaling pathway in diabetic complications | 8 | 0.86156 | 9.2855 | 1.9406E-06 | 0.000048665 |
| hsa05212 | Pancreatic cancer | 7 | 0.6527 | 10.725 | 0.000003386 | 0.000076028 |
| hsa04659 | Th17 cell differentiation | 8 | 0.93118 | 8.5912 | 3.4982E-06 | 0.000076028 |
| hsa04670 | Leukocyte transendothelial migration | 8 | 0.9747 | 8.2077 | 4.9318E-06 | 0.00010048 |
| hsa05145 | Toxoplasmosis | 8 | 0.9834 | 8.1351 | 5.2714E-06 | 0.00010109 |
| hsa04380 | Osteoclast differentiation | 8 | 1.1139 | 7.1817 | 0.000013293 | 0.00022833 |
| hsa04658 | Th1 and Th2 cell differentiation | 7 | 0.80064 | 8.743 | 0.000013307 | 0.00022833 |
| hsa04926 | Relaxin signaling pathway | 8 | 1.1313 | 7.0712 | 0.000014896 | 0.0002428 |

**Table S5:** Docking score of the core TLEO components and core targets

| **SL** | **Protein Name** | **1-Octen-3-yl acetate** | **Geranyl acetate** | **Nerol acetate** | **Linalyl acetate** | **α-Terpineol** | **Hexyl acetate** | **Lavandulol acetate** | **Linalool** | **Terpinen-4-ol** | **1-Octen-3-ol** | **Borneol** | **Lavandulol** |
| --- | --- | --- | --- | --- | --- | --- | --- | --- | --- | --- | --- | --- | --- |
| 1 | **MMP9** | **-5.9** | -5 | -4.8 | -4.8 | -4.8 | **-6** | **-6** | -4.9 | -4.8 | -4.8 | -4.8 | -4.9 |
| 2 | **EGFR** | -5.4 | **-6.9** | **-6.8** | **-6.0** | **-5.9** | -5.1 | **-5.9** | -5.4 | **-6.1** | -5.0 | -4.8 | **-5.6** |
| 3 | **PTGS2** | -4.8 | -5.1 | -5.2 | -5.2 | -5.5 | -5.2 | -5.2 | -5.2 | -5.1 | -4.7 | -5.5 | -5.1 |
| 4 | VCAM1 | -4 | -4.2 | -4.1 | -4 | -4.1 | -4.2 | -4.1 | -4.3 | -4.3 | -4 | -4 | -4.2 |
| 5 | ESR1 | -5.5 | **-6.3** | -5.5 | -5 | -5.5 | -5.4 | -4.8 | -5.5 | -5.5 | -5.5 | -5.4 | -5.1 |
| 6 | PTPRC | -4.8 | -4.7 | -4.6 | -5 | -4.9 | -4.5 | -4.6 | -4.6 | -4.9 | -5 | -4.9 | -5 |
| 7 | ICAM1 | -3.9 | -3.8 | -3.9 | -3.8 | -3.9 | -3.8 | -3.8 | -3.8 | -4 | -3.8 | -3.8 | -3.7 |
| 8 | MMP2 | -5.4 | -5.3 | -5.3 | -5.4 | -5.4 | -5.4 | -5.4 | **-6.0** | -5.3 | -5.3 | -5.2 | -5.5 |
| 9 | MMP1 | -4.8 | -4.5 | -4.5 | -4.6 | -4.3 | -4.6 | -4.7 | -4.3 | -4.7 | -4.6 | -4.6 | -4.6 |
| 10 | SELE | -3.9 | -4.0 | -3.8 | -3.8 | -4.0 | -4.0 | -3.9 | -4.1 | -3.7 | -4 | -3.5 | -3.7 |
| 11 | MAPK14 | -5.3 | -4.7 | -4.7 | -4.6 | -4.7 | -4.6 | -4.8 | -4.6 | -5.2 | -4.7 | -4.6 | -4.6 |
| 12 | MAPK8 | -4.2 | -4.6 | -4.4 | -4.7 | -4.4 | -4.3 | -4.5 | -4.6 | -4.6 | -4.4 | -4.6 | -4.5 |
